# Immune response profiling of patients with spondyloarthritis reveals signalling networks mediating TNF-blocker function in vivo

**DOI:** 10.1136/annrheumdis-2020-218304

**Published:** 2020-12-02

**Authors:** Silvia Menegatti, Vincent Guillemot, Eleonora Latis, Hanane Yahia-Cherbal, Daniela Mittermüller, Vincent Rouilly, Elena Mascia, Nicolas Rosine, Surya Koturan, Gael A Millot, Claire Leloup, Darragh Duffy, Aude Gleizes, Salima Hacein-Bey-Abina, Milieu Intérieur Consortium, Jérémie Sellam, Francis Berenbaum, Corinne Miceli-Richard, Maxime Dougados, Elisabetta Bianchi, Lars Rogge, Milieu Intérieur Consortium, Laurent Abel

**Affiliations:** 1 Immunoregulation Unit, Department of Immunology, Institut Pasteur, Paris, France; 2 Université Paris Diderot, Sorbonne Paris Cité, Paris, France; 3 INSERM U932, Institut Curie, PSL Research University, Paris, France; 4 Bioinformatics and Biostatistics Hub—Département de Biologie Computationelle, Institut Pasteur, USR 3756 IP CNRS, Paris, France; 5 DATACTIX, Paris, France; 6 Institut Pasteur, Translational Immunology Laboratory, Department of Immunology, Paris, France; 7 Clinical Immunology Laboratory, Groupe Hospitalier Universitaire Paris-Sud, Hôpital Kremlin-Bicêtre, AP-HP, Le-Kremlin-Bicêtre, France; 8 UTCBS CNRS UMR 8258, INSERM U1267, Faculté de Pharmacie de Paris, Université de Paris, Paris, France; 9 Sorbonne Université, Service de Rhumatologie, Hôpital Saint-Antoine, AP-HP, Paris, France; 10 Centre de Recherche Saint-Antoine, INSERM UMR_S 938, Paris, France; 11 Paris Descartes University, Rheumatology Department, Cochin Hospital, AP-HP, Paris, France; 12 Unité Mixte AP-HP/Institut Pasteur, Institut Pasteur, Paris, France; 13 INSERM U1153 Clinical Epidemiology and Biostatistics, PRES Sorbonne Paris-Cité, Paris, France

**Keywords:** spondylitis, ankylosing, tumor necrosis factor inhibitors, biological therapy, immune system diseases

## Abstract

**Objectives:**

Antitumour necrosis factor (TNF) therapy has revolutionised treatment of several chronic inflammatory diseases, including spondyloarthritis (SpA). However, TNF inhibitors (TNFi) are not effective in all patients and the biological basis for treatment failure remains unknown. We have analysed induced immune responses to define the mechanism of action of TNF blockers in SpA and to identify immunological correlates of responsiveness to TNFi.

**Methods:**

Immune responses to microbial and pathway-specific stimuli were analysed in peripheral blood samples from 80 patients with axial SpA before and after TNFi treatment, using highly standardised whole-blood stimulation assays. Cytokines and chemokines were measured in a Clinical Laboratory Improvement Amendments (CLIA)-certified laboratory, and gene expression was monitored using nCounter assays.

**Results:**

Anti-TNF therapy induced profound changes in patients’ innate immune responses. TNFi action was selective, and had only minor effects on Th1/Th17 immunity. Modular transcriptional repertoire analysis identified prostaglandin E_2_ synthesis and signalling, leucocyte recirculation, macrophage polarisation, dectin and interleukin (IL)-1 signalling, as well as the nuclear factor kappa B (NF-kB) transcription factor family as key pathways targeted by TNF blockers in vivo. Analysis of induced immune responses before treatment initiation revealed that expression of molecules associated with leucocyte adhesion and invasion, chemotaxis and IL-1 signalling are correlated with therapeutic responses to anti-TNF.

**Conclusions:**

We show that TNFi target multiple immune cell pathways that cooperate to resolve inflammation. We propose that immune response profiling provides new insight into the biology of TNF-blocker action in patients and can identify signalling pathways associated with therapeutic responses to biological therapies.

**Key messages**

**What is already known about this subject?**

- Antitumour necrosis factor (TNF) therapy has revolutionised treatment of many chronic inflammatory diseases, including spondyloarthritis and rheumatoid arthritis. However, TNF inhibitors (TNFi) are not effective in 30%–40% of patients. The immunosuppressive effects of TNF blockers therefore expose a substantial fraction of patients to side-effects, in particular infections, without clinical benefit. Despite the extensive use of TNFi for many years, the biological basis for treatment failure remains unknown.

**What did this study add?**

- We demonstrate that anti-TNF therapy induces profound changes in patients’ innate immune responses, but does not affect Th1/Th17 immunity.
- Modular transcriptional repertoire analysis showed that prostaglandin E_2_ synthesis and signalling, leucocyte recirculation, macrophage polarisation, dectin and interleukin (IL)-1 signalling, as well as the NF-kB transcription factor family are key pathways targeted by TNF blockers in vivo.
- To investigate the concept that the immune status of patients before treatment initiation will define their response to TNFi treatment, we have searched for immunological transcripts that correlate with clinical efficacy of TNF blockers in stimulated immune cells. We found that high expression of molecules associated with leucocyte adhesion and invasion, chemotaxis and IL-1 signalling is correlated with favourable outcome of anti-TNF therapy.

**Key messages**

**How might this study impact on clinical practice or future developments?**

- We have established a robust pipeline to monitor immune responses in patients that can be translated into a clinical setting. We show that immune response profiling can identify signalling pathways associated with therapeutic responses to TNFi. Further studies will assess whether this approach can be used to develop molecular biomarkers to help stratify patients to the most appropriate therapy.

## Introduction

Chronic inflammatory diseases (CID) are challenging illnesses that often strike at a young age and cause lifelong morbidity, representing a considerable burden for the affected individuals and for society. Spondyloarthritis (SpA) is a family of related inflammatory disorders with common pathological and genetic features.^1–3^ Clinical manifestations include spinal (axial) inflammation, peripheral arthritis, enthesitis and extra-articular features such as uveitis, psoriasis and inflammatory bowel disease.^4^

Antitumour necrosis factor (TNF) therapy has proven effective to reduce inflammation and clinical symptoms in SpA; however, little is known about how TNF inhibitors (TNFi) affect immune responses in patients, and TNFi have been associated with infectious complications,^5^ including *Mycobacterium tuberculosis* reactivation.^6–8^

Furthermore, the high rate of non-responsiveness (30%–40%) to TNFi exposes a substantial fraction of patients to side effects without clinical benefit, and it is still not possible to determine which patients will respond to TNFi before treatment initiation.^9–11^ The recent introduction of antibodies-blocking interleukin (IL)-17A has expanded the therapeutic options for axial SpA (axSpA), as well as psoriasis and psoriatic arthritis.^12 13^ It is therefore important to develop tools to guide treatment decisions for patients affected by SpA and other CID, to optimise clinical care and contain healthcare costs.

Here, we investigated the global impact of TNFi on immune responses to microbial or pathway-specific stimuli, with the goal to enhance our understanding of the molecular mechanism of action of TNF blockers in patients with SpA and to identify immunological correlates of responsiveness to TNFi.

## Methods

### Patients

Peripheral blood samples were obtained from 80 biologic-naïve patients fulfilling Assessment of SpondyloArthritis international Society (ASAS) criteria for axSpA,^14 15^ attending the Rheumatology Departments of Cochin or Saint-Antoine Hospitals (Paris, France). A written informed consent has been obtained from each subject.

Patients’ demographics, HLA-B27 status, information regarding symptoms, ongoing treatments, comorbidities and other main clinical features of SpA were recorded on a Case Record Form before and 3 months (D90) after initiation of anti-TNF therapy (see table 1 and online supplemental table 1).

10.1136/annrheumdis-2020-218304.supp1 Supplementary data

**Table 1:** Clinical characteristics of the 80 patients with axial spondyloarthritis (axSpA) included in the study

Characteristic	SpA (n=80)
Female n (%)	25 (31%)
Median (IQR) age at sampling (years)	37 (19–64)
Median (IQR) disease duration (years)	2 (0–33)
HLA-B27 positive n (%)	63 (79%)
Current smokers n (%)	40 (50%)
Median (IQR) C reactive protein (CRP) (mg/L) at baseline	6.06 (0.09–62)
Median (IQR) BASDAI at baseline	49.80 (9.40–90)
Median (IQR) ASDAS at baseline	3.05 (1.13–4.79)
Axial involvement n (%)	80 (100%)
Axial and enthesial involvement n (%)	38 (47.5%)
Radiological sacroiliitis n (%)	48 (60%)
MRI sacroiliitis n (%)	63 (79%)
*TNF blocker*	
Soluble TNF receptor etanercept n (%)	53 (66.25%)
Monoclonal antibody adalimumab n (%)	13 (16.25%)
Monoclonal antibody golimumab n (%)	13 (16.25%)
Monoclonal antibody infliximab n (%)	1 (1.25%)
*Extra-articular manifestations*	
Psoriasis n (%)	16 (20%)
Uveitis n (%)	26 (33%)
IBD (%)	3 (4%)
*Response at D90*	
Median (IQR) CRP (mg/L) at D90	1.95 (0–51.80)
Median (IQR) BASDAI at D90	23.50 (0–78)
Median (IQR) ASDAS at D90	1.44 (0.64–3.45)
Patients with major ASDAS improvement n (%)	20 (25%)
Patients with clinically important improvement ASDAS n (%)	30 (37.5%)
Non-responder ASDAS n (%)	30 (37.5%)
Non-responder ASDAS treated with etanercept n (%)	22 (73.33%) (41.5%)†
Non-responder ASDAS treated with adalimumab n (%)	5 (16.67%) (38.5%)†
Non-responder ASDAS treated with golimumab n (%)	3 (10%) (23.1%)†
Non-responder ASDAS treated with infliximab n (%)	0 (0 %)
Non-responder BASDAI50 n (%)	52 (65%)

Median and IQR or percentages are shown.

*Percentage of total non-responders.

†Percentage of patients treated with the indicated drug.

ASDAS, Ankylosing Spondylitis Disease Activity Score; BASDAI, Bath Ankylosing Spondylitis Disease Activity Index; IBD, inflammatory bowel disease; TNF, tumour necrosis factor.

Primary responsiveness to anti-TNF therapy was based on the Ankylosing Spondylitis Disease Activity Score (ASDAS).^16^ The ‘improvement score’ was calculated as: ASDAS at baseline (D0)—ASDAS at D90. Patients achieving a delta ASDAS <1.1 were classified as non-responders.^16^

Whole-Blood TruCulture Stimulation was performed with TruCulture assays (Myriad RBM, Texas).^17^ Multianalyte profiling of culture supernatants was performed with Luminex xMAP technology (Myriad-RBM, Austin, Texas, USA), gene expression analysis with nCounter Technology (NanoString), with the Human Immunology v2 Gene Expression CodeSet.^18 19^

### Purification of monocytes and in vitro cell stimulation

To generate in vitro derived macrophages, monocytes were isolated from healthy donors and cultured with macrophage colony-stimulating factor (M-CSF) in the presence or absence of TNFi. Cells were polarised towards M1 with LPS (20 ng/mL, Invivogen) and interferon (IFN)-γ (20 ng/mL, Milteny), or towards M2 with IL-4 and IL-13 (20 ng/mL, Miltenyi).

### Data analysis

Quantitative set analysis of gene expression was performed using the R QuSage package.^20^ Differential gene expression was analysed using the LIMMA package^21^; principal component analysis and hierarchical clustering were performed with Qlucore Omics Explorer (Qlucore).

Methods are described in detail in the online supplementary material.

## Results

### TNFi affect immune responses to microbes and stimuli targeting specific immune receptors

**Figure 1:**
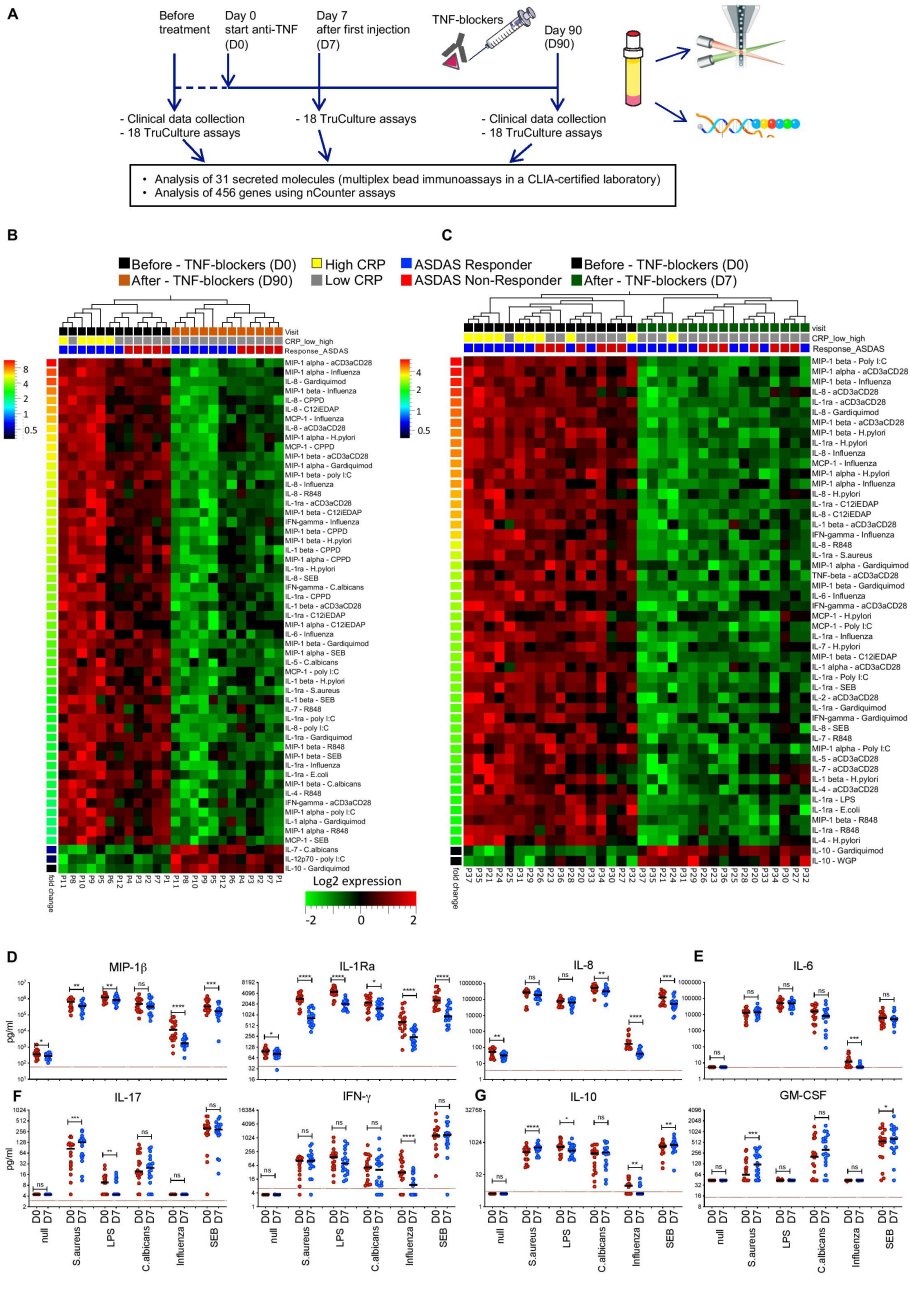
An immunological signature of antitumour necrosis factor (TNF) therapy. (A) Study design. Blood samples were collected from patients with axial spondyloarthritis (axSpA) prior to (D0), 7 days (D7, for a subset of patients), and 3 months (D90) after beginning TNF inhibitors (TNFi) treatment. Clinical efficacy was monitored at D90 according to the current standard of care. (B) The levels of 31 secreted molecules in response to 18 different immune stimuli were compared in samples from 12 patients at D0 (black rectangles) and D90 (orange rectangles). Patients with C reactive protein (CRP) levels >6 mg/L are marked with yellow rectangles, while CRP levels <6 mg/L are indicated with grey rectangles. Patients responding to anti-TNF therapy (delta ASDAS ≥1.1) are marked in blue and non-responders (delta ASDAS <1.1) are marked in red. The heatmap shows the levels of differentially secreted proteins (paired t-test, FDR≤0.05, fold-change ≥2, red indicates higher and green lower levels of protein secretion). Analyte-stimulus combinations were ranked by decreasing fold change (color-code bar, top left); patient IDs are indicated below the heatmaps. (C) The same analysis as in (B) was performed for additional 17 patients with axSpA, sampled at D0 (blue rectangles) and D7 (green rectangles). (D–G) Levels of proteins identified in (C), for 5 representative stimuli and the unstimulated (null) condition, in 17 patients with axSpA at D0 (red) and D7 (blue). Red lines indicate the least detectable dose (LDD) for each assay. P values were calculated using a Wilcoxon matched-pairs test (patients with SpA D0 vs D7) *: p<0.05; **: p<0.01; ***: p<0.001; ****: p<0.0001; ns, not significant. Horizontal black bars indicate the median. Y-axes are log10 or log2 scales. ASDAS, Ankylosing Spondylitis Disease Activity Score; IFN, interferon; IL, interleukin.

We analysed immune responses in patients with axSpA with indications for TNFi treatment (table 1), using whole blood (‘TruCulture’) assays^17^ (figure 1A). We stimulated blood samples from 12 patients with a range of microbial stimuli or signalling agonists, and we measured the levels of 31 secreted molecules (online supplemental tables 3 and 4, online supplemental figure 1A). Three months (D90) after TNFi initiation, the induction of many proinflammatory cytokines and chemokines (such as macrophage inflammatory protein-1beta (MIP-1β), IL-1Ra and IL-8) was reduced in response to various stimuli, indicating that TNFi target intracellular pathways shared by a broad range of immune activators (figure 1B). In contrast, TNFi had no major effects on IL-6, IFN-γ and IL-17 (online supplemental figure 1D), although the Th17 pathway is suggested to be of key importance in SpA pathophysiology.^22^

Only few secreted proteins increased after TNFi therapy. Among these was IL-10 following stimulation with gardiquimod (figure 1B), a selective ligand for TLR7.

These results show that TNFi induce selective changes in patients’ immune responses, mostly detected in the challenged immune system, and not in the resting state (online supplemental figure 1D).

### The effects of TNFi are detected after a single injection and remain stable over time

To determine the early effects of TNFi, we analysed 17 consecutive patients with axSpA 7 days after initiation of TNFi therapy (online supplemental figure 1B). Secretion of proinflammatory mediators was already affected after a single TNFi injection (figure 1C, D and G) and over a broad range of stimuli (online supplemental figure 2A). Production of IL-6, IL-17 and IFN-γ was largely unaffected (figure 1E, F).

The reduction in proinflammatory mediators was maintained at D90 (online supplemental figure 2B, C), demonstrating that the effects of TNFi on immune responses remain stable over time.

### TNF blockers affect key transcriptional networks of innate immune responses

**Figure 2:**
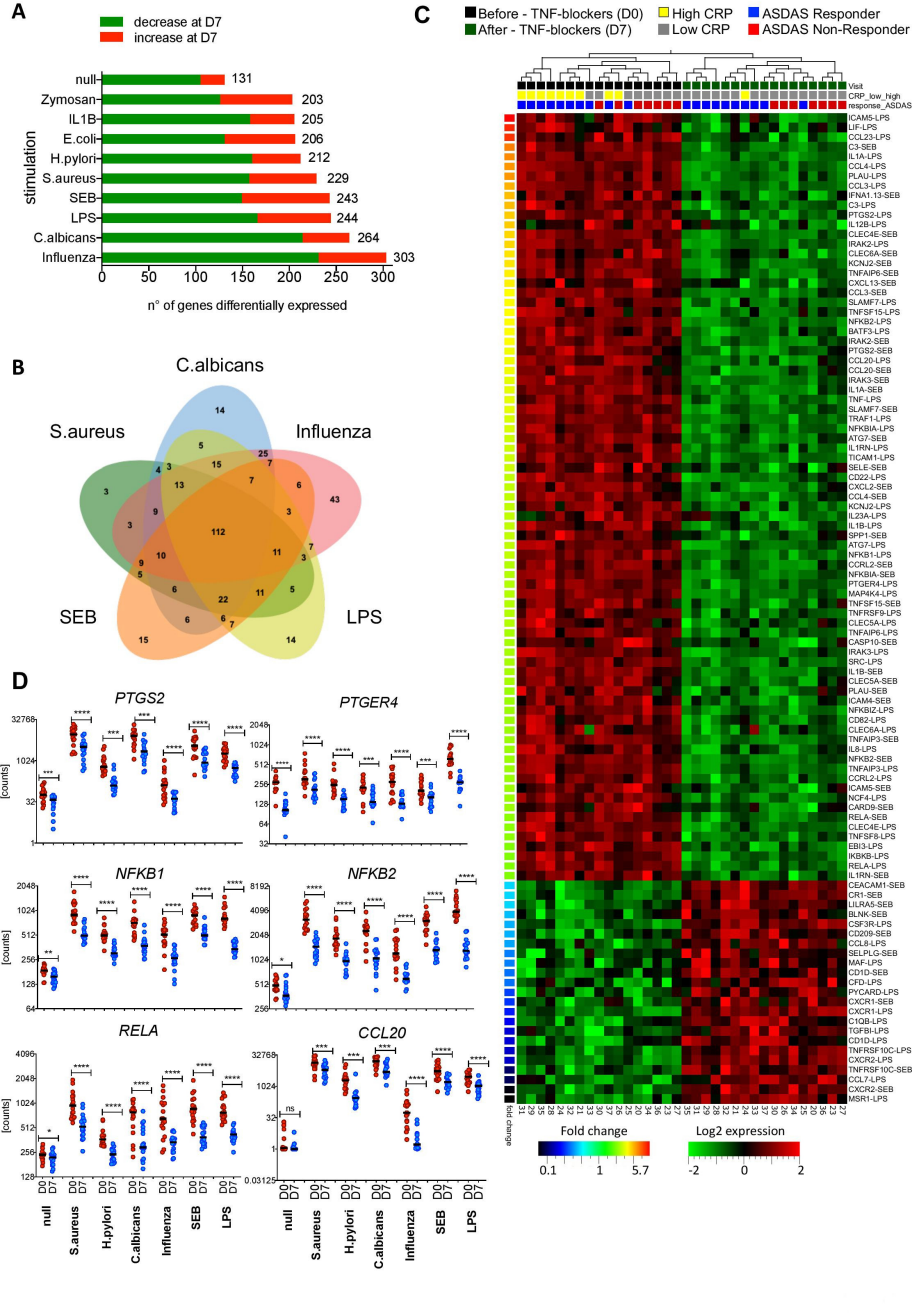
Tumour necrosis factor (TNF) blockers strongly affect key transcriptional networks of innate immune responses. (A) Number of genes differentially expressed in 10 different TruCulture stimulation assays performed at D0 and D7 (17 patients, paired t-test, false discovery rate (FDR)≤0.05). (B) Venn diagram of the genes differentially expressed as in (A), in five representative stimulation conditions. (C) Heatmap showing the genes most affected by TNF inhibitors (TNFi; D0, black rectangles vs D7, green) in lipopolysaccharides (LPS) and staphylococcal enterotoxin (SEB) stimulation conditions. Patients with C reactive protein (CRP) levels >6 mg/L are marked with yellow rectangles, while CRP levels <6 mg/L are indicated with grey rectangles. Patients responding to anti-TNF therapy (delta Ankylosing Spondylitis Disease Activity Score (ASDAS) ≥1.1) at M3 are marked in blue and non-responders (delta ASDAS <1.1) are marked in red. Paired t-test, FDR≤0.005 and fold-difference threshold of ≥2. Gene-stimulus combinations were ranked by decreasing fold change (colour code bottom left bar). (D) Expression levels of *PTGS2*, *PTGER4,* NF-κB family members, and *CCL20* for the unstimulated TruCulture assay and five representative stimuli at D0 (red) and D7 (blue) after initiation of TNFi therapy. P values were determined using a Wilcoxon matched-pairs test (D0 vs D7, *: p<0.05; **: p<0.01; ***: p<0.001; ****: p<0.0001; ns, not significant, n=17). Horizontal black bars indicate the median.

To gain insight into the mechanisms by which TNFi affect immune responses, we analysed the expression of immune-related genes before and at D7 and D90 after TNFi treatment. TNF blockade profoundly altered the transcription of a large number of genes (figure 2A).

The majority of genes differentially expressed after therapy were shared by different stimulation conditions, revealing a ‘core immune response signature’ targeted by TNFi (figure 2B), which included NF-kB genes, such as *NFKB1*, *RELA, NFKB2* and *RELB*, and NF-kB targets, such as *IL1A, IL1B* and *CCL20* (figure 2C and D, online supplemental figure 3A, B). In particular, TNFi strongly downmodulated expression of *PTGS2*, encoding cyclooxygenase (COX-2), the key enzyme in prostaglandin E_2_ (PGE_2_) biosynthesis and *PTGER4* encoding the PGE_2_ receptor EP4 (figure 2D). TNFi-induced downmodulation of *PTGS2* and *PTGER4* did not depend on the NSAID index at baseline (online supplemental figure 4). Consistent with our analysis of secreted proteins (figure 1D), *IL17A*, *IFNG* and *IL6* were largely unaffected (online supplemental figure 3A).

The analysis of patients stratified into responders and non-responders showed that the majority of differentially expressed genes are common to both groups, although a number of genes are uniquely affected in each patient subset (online supplemental table 6 and online supplemental figures 5 and 6).

The effects of TNFi also on gene expression could be measured after a single injection and remained stable over time (online supplemental figure 7A).

To determine if changes in cell populations accounted for these effects, we analysed cell counts at D0 and D90. While leucocyte and monocyte counts remained stable, we observed a modest decrease of neutrophils and increase of lymphocyte counts after TNFi therapy (online supplemental figure 7B).

### Modular transcriptional repertoire analysis reveals multiple mechanisms of TNFi action in vivo

**Figure 3:**
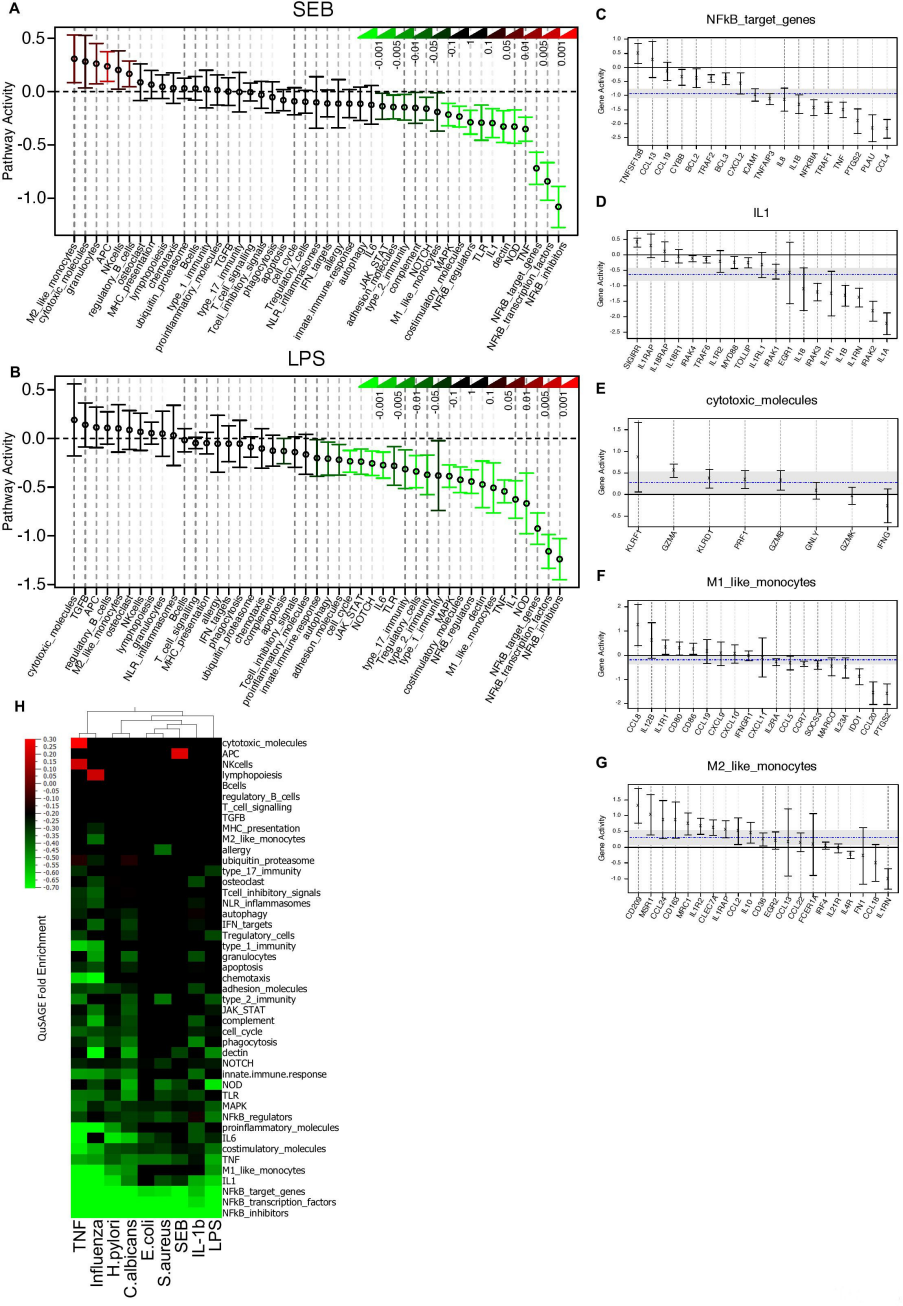
Modular transcriptional repertoire analysis reveals multiple mechanisms of tumour necrosis factor (TNF)-blocker action in spondyloarthritis (SpA). (A, B) Effect of anti-TNF therapy on the activity of 45 gene modules (online supplemental table 5) generated from 456 immune-related genes. Whole-blood cultures were stimulated with SEB (A) or LPS (B). For each gene module, the mean activity fold change and 95% CI are plotted and colour coded according to their FDR-corrected p values (means compared with fold-change zero). CIs overlapping the horizontal dotted line indicate statistically significant increased or decreased module activity at D7 as compared with D0. (C–G) Detailed gene activity in five representative modules with decreased (C, D, E, LPS stimulation) or increased (F, G, SEB stimulation) pathway activity after anti-TNF therapy. The cultures were stimulated with LPS and SEB, respectively. Represented are the mean fold change and 95% CI for individual genes in each module. The horizontal dashed blue line and the grey band indicate the mean differential expression of all genes in the module at D7 versus D0, and the 95% CI. (H) QuSAGE fold enrichment of gene set activity in nine different stimulated cultures at D7 versus D0. For each module, the mean fold change is color coded to indicate increased (red) or decreased (green) module activity. Only changes reaching a significance threshold of FDR≤0.01 are represented. IFN, interferon; IL, interleukin.

The observation that TNFi affected several molecules in the same signalling pathway prompted us to further define the effects of TNFi on immune networks. We compared immune responses at D0 and D7 using Quantitative Set Analysis for Gene Expression (QuSAGE)^20^ (online supplemental table 5). The modules ‘NF-κB transcription factors’ and ‘NF-κB target genes’ were among those most strongly downregulated by TNFi (figure 3A–C and online supplemental table 7), followed by the ‘IL-1/IL-1R’ module (figure 3A, B). Inspection of the individual genes in this module showed downregulation of *IL1A*, *IL1B*, *IRAK2*, *IL1R1* and *IL1RN*, as well as a substantial increase of *SIGIRR*, after TNF blockade (figure 3D).

TNFi therapy also reduced the activity of the ‘dectin’ module (figure 3A, B and online supplemental figure 8A), which groups C-type lectin receptors (CLRs) for *Candida albicans* and other fungi such as Dectin-2 (encoded by *CLEC6A*), or Mincle (encoded by *CLEC4E*) and associated signalling molecules, such as *CARD9*, a molecule involved in antifungal immunity that mediates signals from CLRs to the NF-κB pathway via BCL10.^23^

While gene set activities for most gene modules were reduced by TNFi, we observed increased activity at D7 of the ‘cytotoxic molecules’ module and of the ‘M2-like monocytes’ gene module, while the overall activity of the module ‘M1-like monocytes’ was reduced after TNFi, indicating that TNF blockers may affect monocyte/macrophage polarisation (figure 3).

In particular, we observed an upregulation of the genes encoding surface markers characteristic of regulatory macrophages, such as the mannose receptor *MRC1,* the scavenger receptors *MSR1* and *CD163*, the decoy receptor *IL1R2*, and of *IL10* (figure 3G and online supplemental figure 8B).

Analogous results were obtained at D90 after initiation of TNFi (online supplemental figure 8C), indicating the multiple immune pathways that mediate TNFi function in patients with SpA.

Many of the genes affected by TNFi are expressed in monocytes and macrophages, which prompted us to investigate the roles of these cells in the response to TNFi. We stimulated monocytes from patients with SpA with LPS in the presence or absence of etanercept (Eta), and measured transcript levels before and at different time points after stimulation (online supplemental figure 9). Several of the genes downregulated by etanercept were direct NF-κB target genes, such *NFKBIA*, *TNFAIP3*, *TNFAIP6* or *IL1A* (online supplemental figure 9).

### TNFi skew macrophage polarisation towards an M2 phenotype in vitro

**Figure 4:**
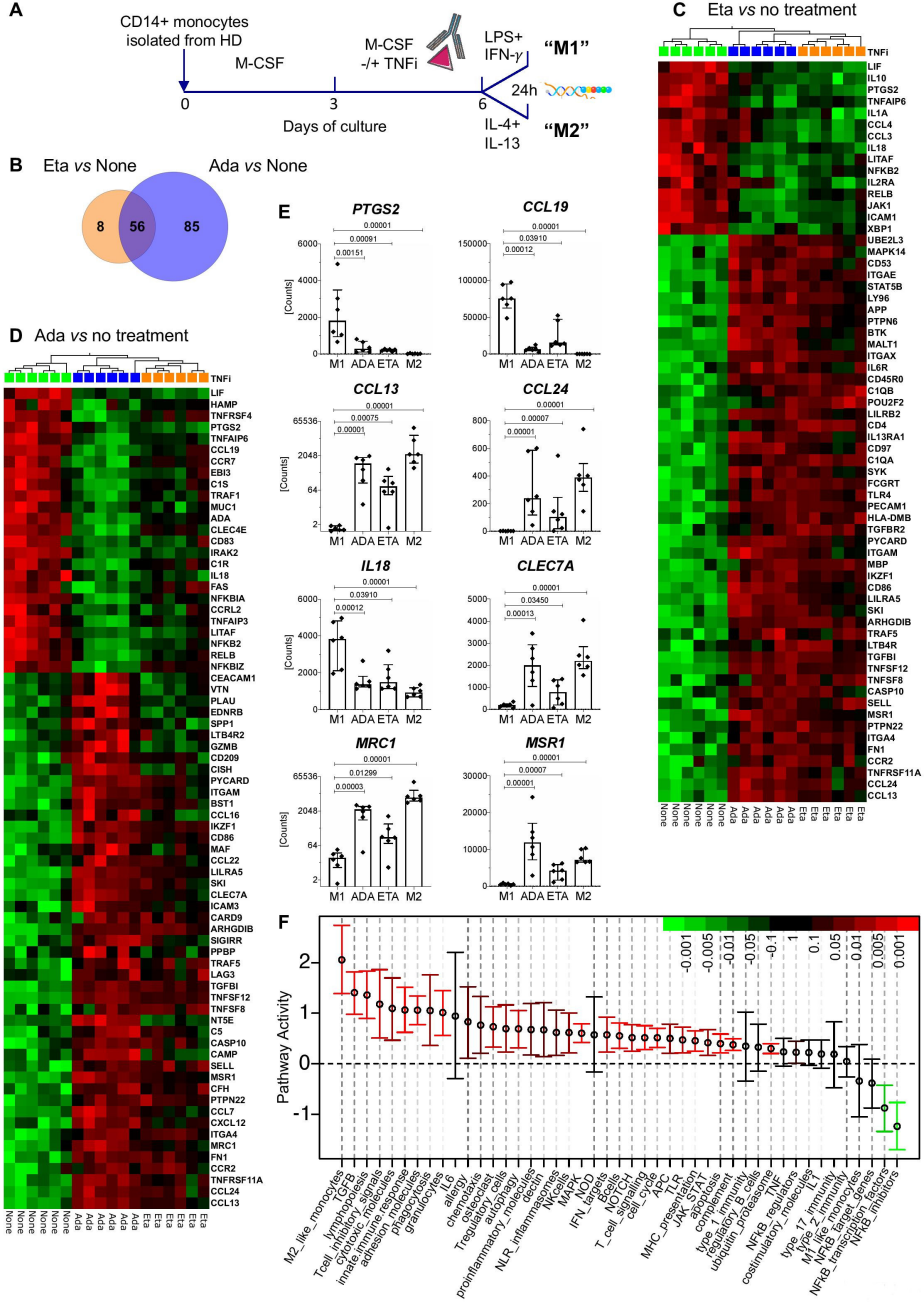
TNF inhibitors (TNFi) have largely overlapping effects on in vitro differentiated M1-type macrophages. (A) Study design. CD14+ cells isolated from healthy donors were differentiated in vitro into macrophages in the presence or absence of etanercept (Eta) or adalimumab (Ada). TNFi were added at day 3 and macrophages were polarised to the M1 subset in the presence or absence of Eta or Ada. Gene expression was analysed with the nCounter Human Immunology v2 panel and with LIMMA (paired sample adjusted p value threshold 0.01). (B) Venn diagram showing the overlap of genes affected by Eta or Ada. Analysis of paired samples with LIMMA, adjusted p value threshold 0.01. (C, D) Heatmaps showing the genes most affected by Eta (orange rectangles) versus no treatment (green rectangles) (C) and Ada (blue rectangles) versus no treatment (D) in macrophages stimulated for 24 hours with LPS and interferon (IFN)-γ (‘M1’ polarisation). (C) Paired t-test, Eta versus no treatment, adjusted p value threshold 0.01. Included are also gene expression levels for Ada-treated samples for the same genes. (D) Paired t-test, Ada versus no treatment and fold-change threshold of ≥2. Included are also gene expression levels for Eta-treated samples for the same genes. Samples were ordered by hierarchical clustering and genes were ranked by decreasing fold change. (E) Shown are the mRNA levels of eight selected genes from (C) and (D) in untreated M1-polarised macrophages (M1), M1 macrophages treated with Ada, M1 macrophages treated with Eta or untreated M2-polarised macrophages (M2). Symbols represent individual data points, boxes the median and whiskers the IQR. Adjusted p values are those of the LIMMA analysis. (F) Effect of Ada on the activity of 45 gene modules (online supplemental table 5) as in figure 3. For each gene module, the mean activity fold change and 95% CI are plotted and color coded according to their FDR-corrected p values compared with zero. Red and green bars indicate statistically significant increased or decreased module activity, respectively, in M1 polarised macrophages treated with Ada versus no treatment.

We then asked whether TNFi affect also macrophage gene expression. As the analysis of tissues is rarely performed in axSpA,^24^ we investigated the effects of two TNFi, etanercept and adalimumab, on in vitro differentiated macrophages (figure 4A). Although the effects of adalimumab on gene expression were stronger in our system, a core of 56 genes was regulated by both TNFi (figure 4B–E).

We noted strong downregulation of M1-macrophages genes such as *IL18* (figure 4C, D and E), while expression of genes associated with M2 macrophages, such *MRC1*, *MSR1* and *CLEC7A* was significantly increased (figure 4E).

TNFi also strongly downmodulated *PTGS2* expression in stimulated M1 macrophages (figure 4E), and affected the mRNA levels of chemokines and their receptors: the expression of *CCL19*, *CCL4* and *CCL3* was downregulated, while *CCL13* and *CCL24* were upregulated by TNFi (figure 4C, D and E). These data are consistent with our results for TNFi treatment in vivo and suggest that TNFi may affect leucocyte recruitment to inflamed joints.

Finally, we confirmed a significant downregulation of NF-κB pathway genes (figure 4C, D and F). These data further support the notion that TNFi affect immune responses by acting on multiple inflammatory pathways and that phagocytic cells are important targets of these effects (figure 4F).

### Immune gene expression associated with therapeutic responses to anti-TNF therapy

**Figure 5:**
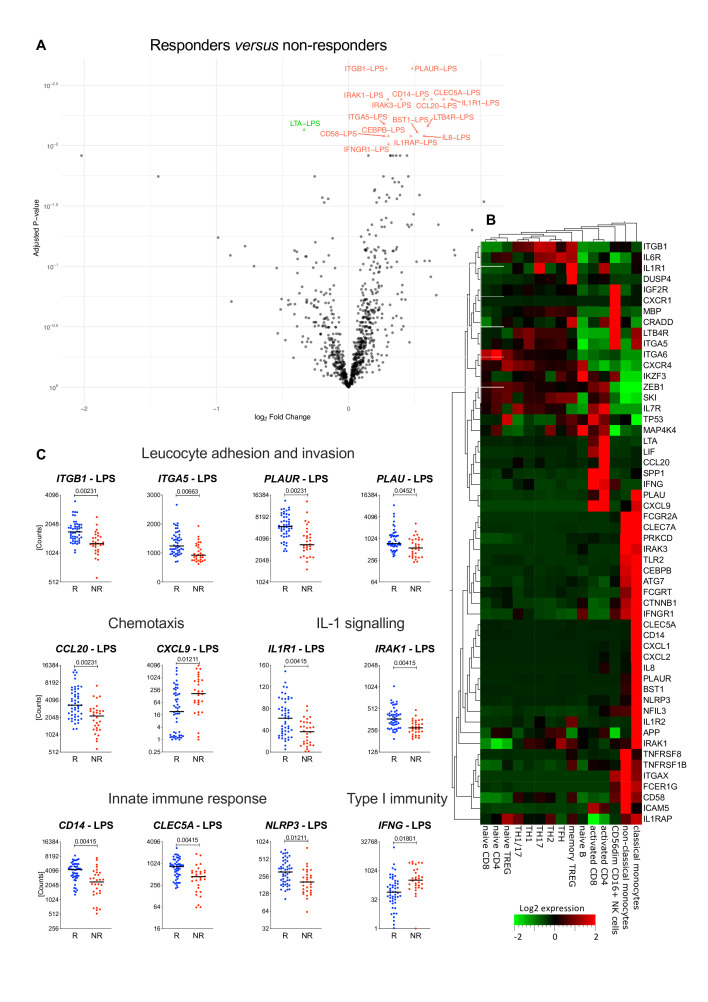
Immune gene expression associated with therapeutic responses to antitumour necrosis factor (TNF) therapy. (A) Volcano plot representation of genes differentially expressed between 50 patients with spondyloarthritis (SpA) responding to anti-TNF therapy and 30 non-responders in whole-blood cultures stimulated with LPS or SEB before initiation of therapy; red triangles: genes higher in responders; green triangle: higher in non-responders (LIMMA analysis, adjusted p value<0.05). Expression levels and fold-change values of the 58 gene-stimulus combinations (corresponding to 55 genes) that are the most differentially expressed between responders and non-responders are reported in table 2. (B). The heatmap shows the expression levels of the differentially expressed genes in different immune cell subpopulations. Gene expression data were extracted from the DICE database (http://dice-database.org/). (C) The expression levels of selected gene-stimulus combinations correlated with treatment response are plotted before treatment initiation (D0). Patients with major or clinically important improvement of disease activity were grouped together as responders and are represented in blue (R, blue, n=50). Non-responders are represented in red (NR, red, n=30). The horizontal black line represents the median. Statistical significance was tested using LIMMA analysis (responders vs non-responders) and adjusted p values are indicated above the graph. IL, interleukin.

Finally, we investigated the correlation between therapeutic responses to TNFi and stimulated immune responses in 80 patients with axSpA, before initiation of anti-TNF therapy. Response to therapy was calculated as the delta ASDAS ‘improvement score’ (ASDAS D0—ASDAS D90).^16 25^ Fifty patients (62.5%) had either a major or a clinically important improvement (‘responders’, delta ASDAS≥1.1), while 30 (37.5%) were non-responders (table 1 and online supplemental table 1). The analysis of whole-blood cultures stimulated with LPS or SEB revealed that 55 genes were differentially expressed between responders and non-responders (table 2 and figure 5A).

**Table 2:** Genes differentially expressed between responders and non-responders to TNFi

Gene ID	Log fold-change (R/NR)	P value (R/NR)	Adjusted P value (R/NR)
*PLAUR*_LPS	0.4816	2.86E−06	0.0023
*ITGB1*_LPS	0.2860	5.29E−06	0.0023
*CD14*_LPS	0.5704	1.78E−05	0.0041
*CCL20*_LPS	0.6264	2.04E−05	0.0041
*IL1R1*_LPS	0.7803	2.48E−05	0.0041
*IRAK1*_LPS	0.2964	3.41E−05	0.0041
*IRAK3*_LPS	0.3977	3.49E−05	0.0041
*CLEC5A*_LPS	0.7180	3.8E−05	0.0041
*ITGA5*_LPS	0.2684	0.0001	0.0066
*LTB4R*_LPS	0.5985	0.0001	0.0069
*LTA*_LPS	−0.3366	0.0001	0.0074
*BST1*_LPS	0.5186	0.0001	0.0077
*IL1RAP*_LPS	0.4707	0.0001	0.0083
*CD58*_LPS	0.2690	0.0001	0.0083
*CEBPB*_LPS	0.2989	0.0001	0.0083
*IL8*_LPS	0.5694	0.0002	0.0083
*IFNGR1*_LPS	0.3022	0.0002	0.0097
*IL1R2*_LPS	0.4411	0.0003	0.0121
*CXCL9*_LPS	−2.0206	0.0003	0.0121
*TNFRSF1B*_LPS	0.3157	0.0003	0.0121
*IL6R*_LPS	0.3360	0.0003	0.0121
*NLRP3*_LPS	0.3896	0.0003	0.0121
*CTNNB1*_LPS	0.1495	0.0003	0.0121
*FCGRT*_LPS	0.3159	0.0003	0.0121
*ITGAX*_LPS	0.3600	0.0003	0.0121
*IFNG*_LPS	−1.4398	0.0005	0.0180
*CXCL1*_LPS	0.4515	0.0006	0.0180
*FCGR2A*_LPS	0.2634	0.0006	0.0180
*ITGA6*_SEB	−0.2569	0.0006	0.0180
*PRKCD*_LPS	0.3330	0.0006	0.0187
*ZEB1*_LPS	0.3487	0.0007	0.0201
*CLEC7A*_LPS	0.3795	0.0007	0.0201
*PECAM1*_LPS	0.4050	0.0008	0.0218
*IRAK1*_SEB	0.1988	0.0009	0.0231
*APP*_LPS	0.1938	0.0010	0.0237
*FCER1G*_LPS	0.2902	0.0011	0.0255
*ICAM5*_SEB	0.5363	0.0011	0.0257
*IL8*_SEB	0.3880	0.0011	0.0257
*PLAUR*_SEB	0.3067	0.0012	0.0270
*IL7R*_SEB	−0.1991	0.0012	0.0270
*IGF2R*_LPS	0.2310	0.0013	0.0270
*IKZF3*_LPS	−0.1544	0.0013	0.0276
*TNFRSF8*_LPS	0.3647	0.0014	0.0276
*NFIL3*_LPS	0.2830	0.0015	0.0290
*LIF*_LPS	1.0229	0.0015	0.0292
*MBP*_LPS	0.2114	0.0016	0.0296
*TP53*_LPS	−0.1846	0.0016	0.0296
*CXCL2*_LPS	0.4914	0.0020	0.0371
*CXCR4*_LPS	0.2833	0.0022	0.0398
*ATG7*_LPS	0.2486	0.0024	0.0412
*CRADD*_SEB	0.3238	0.0025	0.0435
*PLAU*_LPS	0.4759	0.0027	0.0452
*SPP1*_SEB	0.4451	0.0028	0.0452
*SKI*_LPS	0.1760	0.0028	0.0452
*CXCR1*_LPS	0.6786	0.0029	0.0452
*TLR2*_LPS	0.2718	0.0031	0.0471
*MAP4K4*_LPS	0.2504	0.0031	0.0471
*DUSP4*_LPS	0.4570	0.0031	0.0471

To explore if different types of anti-TNF drugs could have an impact on therapeutic responses to TNFi, we compared differential gene expression between responders and non-responders treated with soluble TNFR2 (n=53) to those treated with monoclonal antibodies (n=27). We found a good correlation (R=0.901) for the 55 genes differentially expressed. These data indicate that the type of TNF blockers does not have a major effect on the genes significantly associated with therapeutic responses before treatment (online supplemental figure 10B).

A search of the DICE database^26^ showed expression of these genes in different immune cells, including activated T cells, Treg, Th17 and NK cells (figure 5B). Notably, 29 of the genes were expressed specifically in resting classical or non-classical monocytes (figure 5B). These data suggest that several immune cell populations contribute to determine the efficacy of anti-TNF therapy in patients with SpA.

Among the 55 differentially expressed genes, 15 regulate key steps of leucocyte migration and invasion: these include *PLAU* and *PLAUR*, the integrin subunits *ITGB1*, *ITGA5*, *ITGAX*, and *ITGA6*, and the CD2 ligand *CD58* (figure 5B, C and table 2). The importance of leucocyte recirculation as a determinant of therapeutic responses to TNFi is supported by the observation that several genes encoding chemokines and their receptors, such as *CCL20*, *IL8, CXCL1, CXCL2* and *CXCR1* are expressed at higher levels in cultures from patients with SpA responding to TNFi than in non-responders, while *CXCL9* is expressed at higher levels in non-responders (figure 5B–C, table 2 and online supplemental figure 10). Expression of the receptors for the pro-inflammatory cytokines TNF (*TNFRSF1B*), IL-6 (*IL6R*) and IL-1 (*IL1R1*, *IL1R2* and *IL1RAP*) was also substantially higher in responders than in non-responders, as was expression of the IL-1R-associated kinases *IRAK1* and *IRAK3,* and of *NLRP3*, which controls caspase-1-dependent processing of pro-IL-1β and IL-18. These data indicate that the activation status of the IL-1 signalling pathway may influence responsiveness to TNFi. We also noted substantially higher expression in responders of *CLEC5A* (MDL-1, myeloid DAP12-associating lectin-1), an important mediator of autoimmune inflammation in experimental arthritis models^27^ (figure 5C and table 2).

## Discussion

To investigate immune responses in patients with SpA, we have used highly standardised and robust assays that may be directly translated into a clinical setting. ‘TruCulture’ assays were designed to preserve physiological cellular interactions and capture immune cell activity without introducing sample collection and manipulation variables.^28^ We chose to analyse responses in whole blood, because tissue biopsies cannot be performed routinely in axSpA.

Most of the effects of TNFi could be observed only in stimulated cultures, supporting the notion that TNFi act on activated immune cells, rather than in homeostatic conditions. This may explain the relatively modest changes in gene expression in response to TNFi detected in a recent study of unstimulated PBMCs from patients with axSpA.^29^

Our modular transcriptional repertoire analysis of the stimulation cultures^20^ established a hierarchy of signalling pathways affected by anti-TNF therapy, with potential clinical implications.

We found a strong decrease of proinflammatory molecules produced primarily by innate immune cells, pointing to the importance of these cells in SpA pathogenesis. The decreased activity of the NF-κB module underlines the major role of these factors in mediating TNF-blocker functions. However, TNF blockade had only minor effects on the expression and secretion of IL-6, contrary to what observed in RA patients.^30^ These data suggest that this cytokine may be more relevant to RA, but less to SpA pathogenesis, consistent with the limited therapeutic efficacy of IL-6-blockade in SpA.^31^

We observed downregulation of the classical, M1-like module and an increase of the non-classically activated, M2-like monocyte gene module activity, consistent with the finding that TNFi can expand a cell population with a M2 macrophage-like appearance in vivo and in vitro.^32 33^ Analysis of the effects of TNFi in vitro provided direct evidence that TNFi act directly on macrophage polarisation. These results are consistent with a previous study performed with in vitro differentiated macrophages from patients with rheumatoid arthritis (RA).^34^ M2 macrophages, characterised by expression of IL-10, high levels of scavenger and mannose receptors, *IL1R2* and *IL1RN*, are implicated in the resolution of inflammation and orchestrate tissue repair and remodelling.^35 36^ Polarisation of monocytes/macrophages towards a M2-like profile may be an additional mechanism by which TNF blockers act on the immune system to regulate inflammatory responses^37^ and could also explain the increased risk of opportunistic infections observed for patients treated with TNFi, in particular *M. tuberculosis*.^38^

TNFi strongly downregulated expression of *PTGS2,* the key enzyme in prostaglandin E_2_ (PGE_2_) biosynthesis and target of non-steroidal anti-inflammatory drugs, the first-line treatment of SpA. PGE_2_ is an important early mediator of enthesitis, the hallmark of SpA^39^ and COX-2 inhibition may be an important mechanism of TNFi therapeutic action in this disease. PGE_2_ induces vasodilation, which may facilitate neutrophil recruitment into the entheseal compartment.^39^ We also found that expression of the PGE_2_ receptor *PTGER4* (EP4) was downregulated by TNFi. Signalling through EP4 upregulates IL-23R expression promoting human Th17 cell development,^40^ and suppresses disease progression in an experimental mouse model of autoimmune encephalomyelitis.^41^ Of note, *PTGER4* has been associated with SpA susceptibility, as have been *NFKB1* and *CARD9,* 42 also strongly downregulated by TNFi. Collectively, these data provide evidence that TNFi target the expression of genes closely linked to SpA pathogenesis.

Our findings suggest that TNFi target several immune cell pathways that cooperate to control inflammation. Targeting PGE_2_ biosynthesis via *PTGS2* downregulation is of particular relevance for enthesitis, a critical early pathogenic feature of spondyloarthitis, while shifting the balance of macrophages from a proinflammatory phenotype to a proresolving phenotype is important for the resolution of synovitis. MDL-1/CLEC5A was among the most strongly downregulated molecule after TNFi therapy. Dengue virus-mediated activation of MDL-1/CLEC5A can trigger potent induction of TNF, IL-6 and IL-1β and NLRP3 inflammasome activation and shock.^43 44^ MDL-1/CLEC5A is also expressed in synovial tissue from RA patients and MDL-1/CLEC5A blockade reduced tissue inflammation and bone erosion in experimental arthritis models.^27^ Reduction of MDL-1/CLEC5A expression by TNFi may result in inhibition of bone erosion and inflammatory cytokine production in SpA.

The involvement of multiple pathways in TNF-blocker functions could also explain the difficulties in identifying a genetic marker for treatment response to TNFi.^45^ We could not identify a single gene whose expression correlates with responsiveness to TNFi, but rather a set of genes. A limitation is that our study focused on a predefined panel with 594 genes. Genome-wide studies may be necessary to identify unique molecular biomarkers. Nevertheless, our data suggest that high expression of molecules associated with leucocyte invasion and migration as well as IL-1 signalling in stimulated immune cells predisposes to favourable outcome of anti-TNF therapy. Furthermore, this study was performed in patients from France and should be replicated in an independent cohort from different genetic and environmental backgrounds, to support the translational value of our findings.

In conclusion, we suggest that immune response profiling of patients is a powerful approach to define the mechanism of action of biological drugs and may be a useful strategy to establish objective criteria guiding treatment decisions.
