# Supplementary material for: Immune response profiling of patients with spondyloarthritis reveals signalling networks mediating TNF-blocker function in vivo
Source: Ann Rheum Dis. 2020 Dec 2;80(4):475–86. doi: 10.1136/annrheumdis-2020-218304 (PMC7958106; doi:10.1136/annrheumdis-2020-218304)
Supplement: Supplementary data [file annrheumdis-2020-218304supp001.pdf]

Supplementary Material for:

Immune response profiling of spondyloarthritis patients reveals signaling networks mediating TNF-blocker function *in vivo*

Silvia Menegatti[1,2,†], Vincent Guillemot[3], Eleonora Latis[1,2], Hanane Yahia-Cherbal[1,2], Daniela Mittermüller[1], Vincent Rouilly[4], Elena Mascia[1], Nicolas Rosine[1,2], Surya Koturan[1,2], Gael A. Millot[3], Claire Leloup[1], Darragh Duffy[5], Aude Gleizes[6,7], Salima Hacein-Bey-Abina[6,7], Milieu Intérieur Consortium[‡], Jérémie Sellam[6,7], Francis Berenbaum[6,7], Corinne Miceli-Richard[1,8,9], Maxime Dougados[8,9,10], Elisabetta Bianchi[1,9], Lars Rogge[1,9*]

Supplementary Methods

Patients

Peripheral blood samples were obtained from 80 consecutive patients with a definitive diagnosis of axial spondyloarthritis (axSpA) attending the Rheumatology Department of Cochin Hospital or the Rheumatology Department of Saint-Antoine Hospital (Paris, France). This study fulfills the current Good Clinical Practice Guidelines and a clinical protocol to analyze peripheral blood from SpA patients before and after therapy with TNF-blockers has been accepted by ethical committees (Comité de Protection des Personnes Ile de France III; Référence CPP: n° AT-100) and Institut Pasteur (Projet de recherché clinique n° 2011-32). The project has been approved by the "comité consultatif sur le traitement de l'information en matière de recherche dans le domaine de la santé (CCTIRS, Référence DGRI CCTIRS MG/CP°2012.035), as well as the "Commission Nationale de l'Information et des Libertés" (CNiL; Project "du genotype à la physiopathologie dans les spondylarthropathies, analyse de l'axe IL-23/Th17 chez les patients traités par un anti-TNF"; Décision DR-2013-080). A written informed consent, in compliance with the applicable regulatory and ethical requirements, has been obtained from each subject. All patients met assessment of spondyloarthritis international society (ASAS) criteria for axSpA.[1, 2] Blood was collected from each participant at days 0, 7 and/or 90 after initiation of anti-TNF therapy.

<u>Inclusion criteria</u>

- Patients aged over 18 and under 65 years

- Compliance with criteria established by the "Assessment of SpondyloArthritis international Society" (ASAS, http://www.asas-group.org/)

Exclusion criteria:

- Other spinal disease clearly defined (e.g. discarthrosis);
- History of any biotherapy;
- It is possible to include patients that have received corticosteroid treatment, with the condition that the therapy is stable for at least 4 weeks at the moment of inclusion, and with a dose inferior to 10 mg prednisone.
- patient with active IBD or ongoing uveitis
- patients with psoriatic involvement more than 10% of the skin surface.
- Pregnancy
- History or current disorders which might interfere with the validity of the informed consent and/or prevent an optimal compliance of the patient to the cohort (e.g. alcoholism, psychological disorders).
- No affiliation with a social security scheme
- Person deprived of liberty by judicial or administrative decision, person subjected to a legal protection measure

The first 12 patients were recruited and analyzed during 2015. Recruitment of the subsequent patients was between 2016 and 2018. Patients' demographics, HLA-B27 status, information regarding evaluation of symptoms (including duration of morning stiffness, pain or swelling in peripheral joints and back pain), ongoing treatments (e.g. analgesics, NSAIDs, DMARDs, physiotherapy), co-morbidities with a specific check-list including in particular cardiovascular and malignant diseases, and other main clinical features of spondyloarthritis (e.g. acute anterior uveitis, psoriasis, inflammatory bowel disease, enthesitis, peripheral articular involvement) were recorded on a Case Record Form before and 3 months after initiation of anti-TNF therapy (see **Table 1 and online supplementary Table 1**). Axial, peripheral or enthesial presentation was clinically assessed.

The Ankylosing Spondylitis Disease Activity Score (ASDAS), the Bath Ankylosing Spondylitis Disease Activity Index (BASDAI), erythrocyte sedimentation rate, C-reactive protein, cholesterol (HDL, LDL) and complete blood count were collected before and 3 months after initiation of anti-TNF therapy. C-Reactive Protein (CRP) levels were measured using the high-sensitivity test (hs-CRP test). Radiological evaluation (including plain X-rays and MRI of the spine and the pelvis) was collected systematically for each patient at baseline and at different times after the beginning of the biotherapy.

Definition of Disease Activity and Response to anti-TNF therapy

The criteria for determining disease activity and primary responsiveness to anti-TNF therapy based on the Ankylosing Spondylitis Disease Activity Score (ASDAS) have been described previously.[3, 4] ASDAS-CRP was calculated at baseline (ASDAS D0) and 3 months after initiation of anti-TNF therapy (ASDAS D90). To assess the clinical response to anti-TNF therapy the "improvement score" (delta ASDAS = ASDAS D0 - ASDAS D90) was calculated. Delta ASDAS ≥2 defines a major improvement (responders), delta ASDAS ≥1.1 defines a clinically important improvement (partial responders) and patients achieving a delta ASDAS < 1.1 were classified as non-responders.[3, 4]

Whole-Blood TruCulture Stimulation

TruCulture tubes (Myriad RBM, Texas) are whole-blood stimulation systems consisting in syringe-based medical devices containing the indicated stimulus resuspended in 2 ml of buffered media.[5] Control tubes with no stimulants to assess background levels of genes and mediators of interest were included for each patient at each time point. TruCulture systems were manufactured in accordance with EN ISO 13485 (Medical Device Directive) standards, at EDI GmbH (Reutlingen, Germany), a subsidiary of Myriad RBM (Austin, TX, USA). All TruCulture tubes used in this study were prepared in the same batch, using the same lot of stimuli, and stored at -20°C until use. We performed whole blood stimulation experiments exactly as described previously.[5]

Multi-analyte Profiling

Supernatants from whole-blood stimulation systems were analyzed with Luminex xMAP technology by Myriad-RBM (Austin, TX, USA) as described.[5]

RNA Extraction

Total RNA was extracted from TruCulture cell pellets lysed in Trizol LS and stored at -80°C. Tubes containing cell lysate were thawed on ice 30 minutes before processing, vortexed twice for 5 min at 2000 rpm to complete thawing and RNA release and centrifuged (3000 x g for 5 min at 4°C) to pellet the cellular debris generated during the Trizol lysis. Total RNA was isolated according to a protocol provided by the supplier (Sigma-Aldrich).

RNA Quality Assessment

RNA concentration was estimated using Qubit RNA HS Assay Kit (Life Technologies, USA) according to the protocol provided by the manufacturer. RNA quality was assessed using an Agilent 2100 Bioanalyzer (Agilent Technologies). The RNA Integrity Number (RIN) was

determined using the LabChip System software and all samples with a RIN > 6 were processed for gene expression analysis.

Gene Expression Analysis with nCounter Technology

The nCounter system, a hybridization-based multiplexed assay, was used for the digital counting of transcripts using protocols provided by the supplier (NanoString). Briefly, 100 ng of total RNA from each sample was hybridized according to manufacturer's instructions with the Human Immunology v2 Gene Expression CodeSet, which contains 594 endogenous gene probes, 8 negative control probes (NEG A to NEG H) and 6 positive control probes (POS A to POS F) designed against six *in vitro* transcribed RNA targets at a range of concentrations (from 128fM to 0.125fM). Data collection was carried out in the nCounter Digital Analyzer at the highest standard data resolution (555 fields of view (FOV) collected per flow cell).

We used in total three different batches of the nCounter XT formulation. To correct for a potential batch effect, the expression level of 24 randomly selected RNA samples was measured with the three batches to calculate the calibration factor.

Quality Control of the NanoString Data

Each sample was analyzed in a multiplexed reaction including eight negative probes and six serial concentrations of positive control probes. Quality control consisted of checking the field of view counted (flag if < 0.75), binding density (flag if not in 0.05 − 2.75 range), linearity of positive controls (flag if $R^2 < 0.9$), and limit of detection for positive controls (flag if 0.5fM positive control < 2 standard deviation (SD) above the mean of the negative controls). Negative control analysis was performed to determine the background for each sample. Of note, we excluded three negative control probes (NEG B, NEG F, NEG H), for which we observed variable expression probably due to cross-reaction with bacterial nucleic acid present in two of the TruCulture stimulation systems (*S. aureus* and SEB). nSolver analysis software (version 3.0, NanoString) and R Software (version 3.3.3), NanoStringQCPro (version 1.12.0), NormqPCR (version 1.26.0) packages) were used for quality control and data normalization.

Normalization of the NanoString Data

A first step of normalization using the internal positive controls permitted correction of potential sources of variation associated with the technical platform (e.g. hybridization, purification, or binding efficiency). To do so, the geometric mean of the positive probe counts was calculated for each sample. The scaling factor for a sample was defined as: (average of all the sample geometric means) / (geometric mean of the considered sample). For each sample, we multiplied all gene counts by the corresponding scaling factor. Next, the

background noise, defined as the mean + 2 SD across the five negative probe counts, was subtracted from each gene in a sample. Finally, to normalize for differences in RNA input we used the same method as in the positive control normalization, except that geometric means were calculated over three housekeeping genes (EEF1G, HPRT1 and TBP). These genes were selected using geNorm method [6], an established approach for identification of stable housekeeping genes, from the 15 candidate genes included in the CodeSet. The impact of anti-TNF treatment on the expression level of these housekeeping genes was also evaluated and none of them were affected by TNFi in patient samples.

Gene Filtering

The Human Immunology v2 gene CodeSet contained a total of 594 probes (15 correspond to housekeeping genes), of which 456 were included in downstream analysis after removing probes mapping to multiple genes or aligning to polymorphic regions with greater than two SNPs (9 probes) and probes with low counts (114 probes). Probes mapping to multiple locations and aligning to polymorphic regions with more than two SNPs were excluded from the analysis as described.[7]

We estimated the background level for each sample as the mean plus 2 standard deviations of the five negative probes counts, excluding NEG B, NEG F and NEG H for which we observed significant differences in counts between conditions as previously explained. We defined as 30 counts the highest background level across all the genes in the different stimulations. In order to easily identify genes that were low in high proportions in a given condition, we calculated for each gene in each condition the percentage of samples with expression below the background (30 counts). We removed 114 genes which expression was below the background level in more than 80% of samples in one condition. A condition was considered a given stimulus at a given time point before or after anti-TNF treatment (D0, D7, D90).

Design of gene modules

We generated 45 gene modules by grouping genes included in the immunology_v2 panel according to the Molecular Signatures Database (MSigDB) annotation (http://software.broadinstitute.org/gsea/msigdb)[8] and manual curation from published literature (see **online supplementary Table 5**). Each gene module contains a minimum of three genes, and the same gene can be included in different modules.

Quantitative set analysis of gene expression

We used quantitative set analysis of gene expression (QuSAGE) to identify differences in gene modules by quantifying gene-module activity using a probability density function.[9]

The analysis was performed using R Bioconductor package v2.6.1. As compared to other gene set enrichment analysis methods, QuSAGE improves power by accounting for inter-gene correlations and quantifies gene-module activity with a complete probability density function (PDF). From this PDF, P values and confidence intervals can be easily extracted.

To generate heatmaps representing QuSAGE fold-enrichment of gene sets in the different stimulated cultures, only changes reaching a significance threshold of FDR ≤ 0.01 were represented. When this threshold was not reached for a given module in a specific culture, the value of 0 was assigned to the fold-change, to reflect no statistically significant change.

Venn diagram

The Venn diagram was generated using the web application jvenn (http://genoweb.toulouse.inra.fr:8091/app/example.html).

Purification of PBMCs and *in vitro* cell stimulation

Peripheral blood mononucleated cells (PBMCs) were isolated from fresh blood samples by gradient separation on Ficoll density gradient centrifugation (Lymphocyte separation medium, Eurobio, France) as described previously.[10] Monocytes were purified by magnetic cell sorting using anti-CD14 monoclonal antibody (mAb)-coated beads as recommended by the manufacturer (Miltenyi Biotec). The purity of monocytes was over 97% as verified by flow cytometry (LSR II, BD Biosciences). CD14+ cells were plated in 48-well plates at a final concentration of 1 x 10^6 PBMCs per ml and cultured for different times in pre-warmed Roswell Park Memorial Institute (RPMI) 1640 medium (Invitrogen) not supplemented with fetal calf serum, nor antibiotics. Untreated cells were immediately lysed in RLT buffer (Qiagen) with 1% β-mercaptoethanol to form the naïve subset and snap frozen for RNA extraction at a later date. All the rest of the monocytes were incubated or not with the soluble receptor etanercept (gift from Rheumatology Hardy B Unit of Cochin Hospital (Paris, France)) at a concentration of 10 µg/ml for 10 minutes at 37°C [11] prior to the stimulation for various times with lipopolysaccharide (LPS, 20 ng/mL) from Escherichia coli (LPS, Invivogen). Cells were harvested after 15, 30, 60, 120 and 240 minutes of stimulation for analysis of mRNA expression. Cultured monocytes were lysed directly in RLT buffer (Qiagen) with 1% β-mercaptoethanol and homogenized by pipetting. mRNA was isolated using a RNeasy Micro kit (Qiagen) and analyzed with the nCounter Human Immunology v2 Gene Expression CodeSet.

Culture of Monocyte-Derived Macrophages

Monocytes were isolated from peripheral blood of six healthy donors using CD14 microbeads (Miltenyi Biotec) and cultured for 3 days in RPMI-Glutamax medium (Gibco)

supplemented with antibiotics (penicillin and streptomycin) and 10% FCS in presence of 50 ng/ml M-CSF (Miltenyi Biotec). Monocyte-derived macrophages were subsequently cultured for three additional days in RPMI with M-CSF in presence or absence of etanercept or adalimumab (gifts from Rheumatology Hardy B Unit of Cochin Hospital (Paris, France)) at a concentration of 10 µg/ml, and then polarized for 24h towards the M1 subset with LPS (20 ng/mL, Invivogen) and IFN-γ (20 ng/ml, Milteny Biotec), or towards the M2 subset with IL-4 and IL-13 (both 20 ng/ml, Miltenyi Biotec). M1- and M2-macrophages were lysed in RLT buffer (Qiagen) with 1% β-mercaptoethanol and homogenized by pipetting. mRNA was isolated using a RNeasy Micro kit (Qiagen) and analyzed with the nCounter Human Immunology v2 Gene Expression CodeSet as described above.

Gene expression analysis for correlation to therapeutic responses

Using baseline clinical parameters (collected before the initiation of anti-TNF therapy), and baseline (D0) NanoString gene expression for LPS and SEB stimulations, differential gene expression analysis was performed to correlate therapeutic responses to TNFi in 80 axSpA patients, according to the delta ASDAS score.

Prior to the differential expression analysis, the NanoString gene expression dataset composed from LPS and SEB stimulations was filtered based on level of expression and pattern of expression. Lowly expressed genes were discarded when their normalized median count was below 30 counts in LPS and SEB stimulation conditions at D0 (R Software v3.3.3, dplyr v0.7.4).

We analyzed differential gene expression between the stimulation cultures from the 50 responders and 30 non-responders using the LIMMA package.[12] with an FDR correction for multiple testing. Age, sex, smoking history, B27 status, comorbidities and type of TNF inhibitor were included as covariates in the analysis. Genes were considered as differentially expressed when their adjusted p-values were lower than 0.05. The differentially expressed genes are reported in **Table 2** with their log Fold-Change, P-values and adjusted P-values.

Statistical analysis

Unless otherwise indicated, horizontal bars represent the median. Statistical tests were two-sided and are specified in figure legends. Differences were considered to be significant when $P < 0.05$. Multiple testing corrections were applied where appropriate. Dot-plot graphs were compiled with GraphPad Prism v.7.0.

Principal component analysis (PCA) and agglomerative hierarchical clustering were performed with Qlucore Omics Explorer, version 3.6 (Qlucore). Before applying PCA and agglomerative hierarchical clustering, the variables (proteins or mRNA expression levels) were log-transformed, mean-centered per donor, and scaled to unit variance.

REFERENCES

1. Rudwaleit M, Landewe R, van der Heijde D*, et al.* The development of Assessment of SpondyloArthritis international Society classification criteria for axial spondyloarthritis (part I): classification of paper patients by expert opinion including uncertainty appraisal. Ann Rheum Dis. 2009 Jun; 68(6):770-776.
2. Rudwaleit M, van der Heijde D, Landewe R*, et al.* The Assessment of SpondyloArthritis International Society classification criteria for peripheral spondyloarthritis and for spondyloarthritis in general. Ann Rheum Dis. 2011 Jan; 70(1):25-31.
3. Machado P, Landewe R, Lie E*, et al.* Ankylosing Spondylitis Disease Activity Score (ASDAS): defining cut-off values for disease activity states and improvement scores. Ann Rheum Dis. 2011 Jan; 70(1):47-53.
4. Machado PM, Landewe R, Heijde DV*, et al.* Ankylosing Spondylitis Disease Activity Score (ASDAS): 2018 update of the nomenclature for disease activity states. Ann Rheum Dis. 2018 Oct; 77(10):1539-1540.
5. Duffy D, Rouilly V, Libri V*, et al.* Functional Analysis via Standardized Whole-Blood Stimulation Systems Defines the Boundaries of a Healthy Immune Response to Complex Stimuli. Immunity. 2014 Mar 20; 40(3):436-450.
6. Vandesompele J, De Preter K, Pattyn F*, et al.* Accurate normalization of real-time quantitative RT-PCR data by geometric averaging of multiple internal control genes. Genome Biol. 2002 Jun 18; 3(7):RESEARCH0034.
7. Urrutia A, Duffy D, Rouilly V*, et al.* Standardized Whole-Blood Transcriptional Profiling Enables the Deconvolution of Complex Induced Immune Responses. Cell Rep. 2016 Sep 06; 16(10):2777-2791.
8. Liberzon A, Birger C, Thorvaldsdottir H*, et al.* The Molecular Signatures Database (MSigDB) hallmark gene set collection. Cell Syst. 2015 Dec 23; 1(6):417-425.
9. Yaari G, Bolen CR, Thakar J*, et al.* Quantitative set analysis for gene expression: a method to quantify gene set differential expression including gene-gene correlations. Nucleic Acids Res. 2013 Oct; 41(18):e170.
10. Coffre M, Roumier M, Rybczynska M*, et al.* Combinatorial control of Th17 and Th1 cell functions by genetic variations in genes associated with the interleukin-23 signaling pathway in spondyloarthritis. Arthritis Rheum. 2013 Jun; 65(6):1510-1521.
11. Mitoma H, Horiuchi T, Tsukamoto H*, et al.* Mechanisms for cytotoxic effects of anti-tumor necrosis factor agents on transmembrane tumor necrosis factor alpha-expressing cells: comparison among infliximab, etanercept, and adalimumab. Arthritis Rheum. 2008 May; 58(5):1248-1257.
12. Ritchie ME, Phipson B, Wu D*, et al.* limma powers differential expression analyses for RNA-sequencing and microarray studies. Nucleic Acids Res. 2015 Apr 20; 43(7):e47.
13. Covert MW, Leung TH, Gaston JE*, et al.* Achieving stability of lipopolysaccharide-induced NF-kappaB activation. Science. 2005 Sep 16; 309(5742):1854-1857.

Supplementary Figure 1

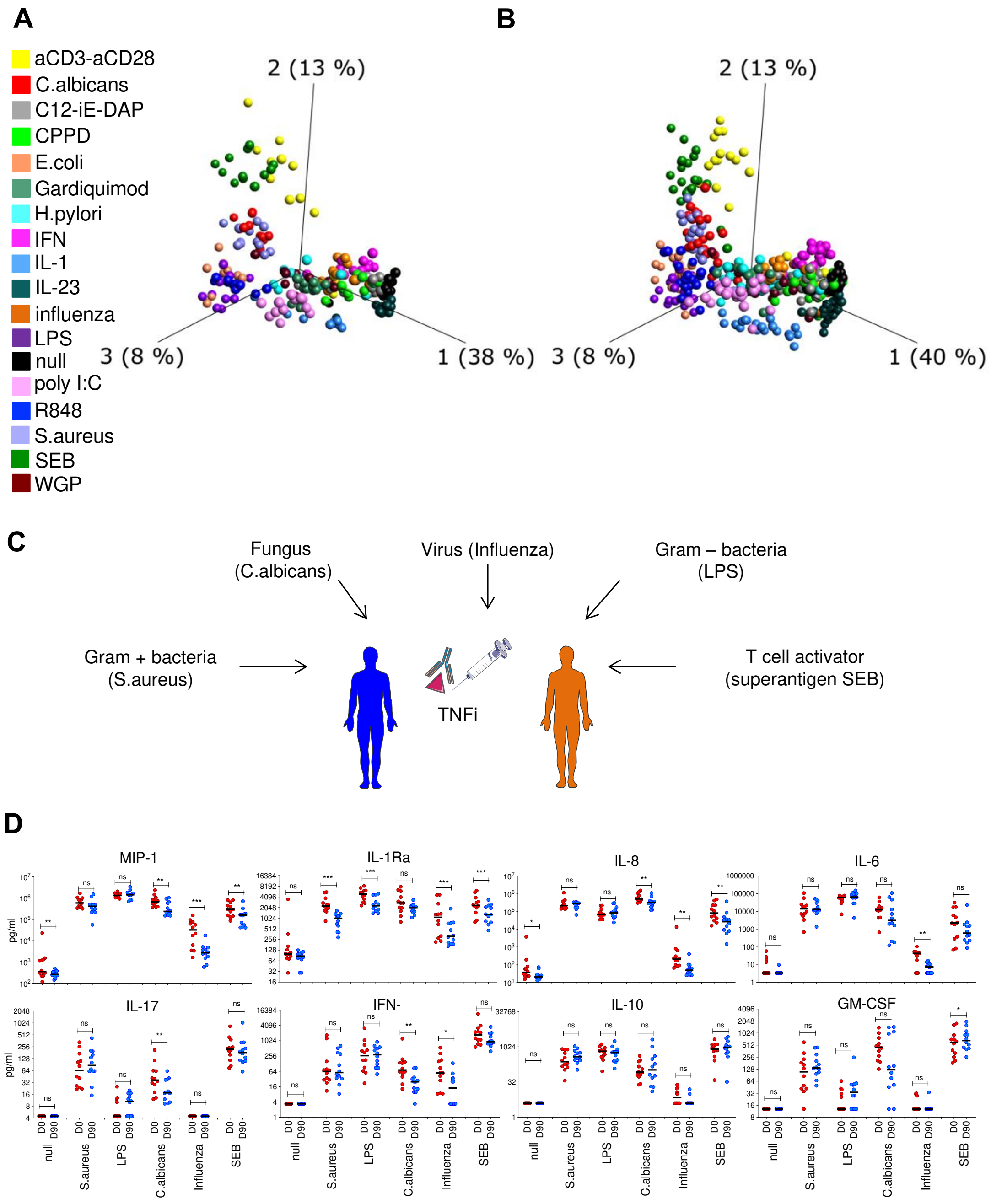

Supplementary Figure 1. Effects of different stimuli on protein signatures.

(**A**) Principal component analysis (PCA) was performed on the secreted protein data obtained from 12 patients before initiation of anti-TNF therapy (D0), measured in 18 different whole blood stimulations. Each filled circle represents a stimulated sample. Although the samples cluster by stimulation, some stimuli largely overlap, reflecting the activation of common signaling pathways. Values for each of the 31 analytes were centered to mean = zero and scaled to unit variance. (**B**) PCA was performed on the secreted protein data obtained from additional 17 patients at D0. The overall PCA structure of this cohort is similar to the one in (A). (**C**) Shown are the representative stimuli selected for further analysis of patient profiles before and after initiation of anti-TNF therapy: S. aureus (a gram-positive bacteria), C. albicans (a yeast), influenza virus, Lipopolysaccharide (LPS) and Staphylococcal enterotoxin B (SEB), a superantigen triggering T cell activation. (**D**) Plots (as in **Fig. 1**) indicate the levels of differentially secreted proteins for 5 representative stimuli and the unstimulated (null) condition, in 12 patients before (D0, in red) and 90 days after (D90, in blue) initiation of anti-TNF therapy (identified as described in **Fig. 1B**).

Supplementary Figure 2

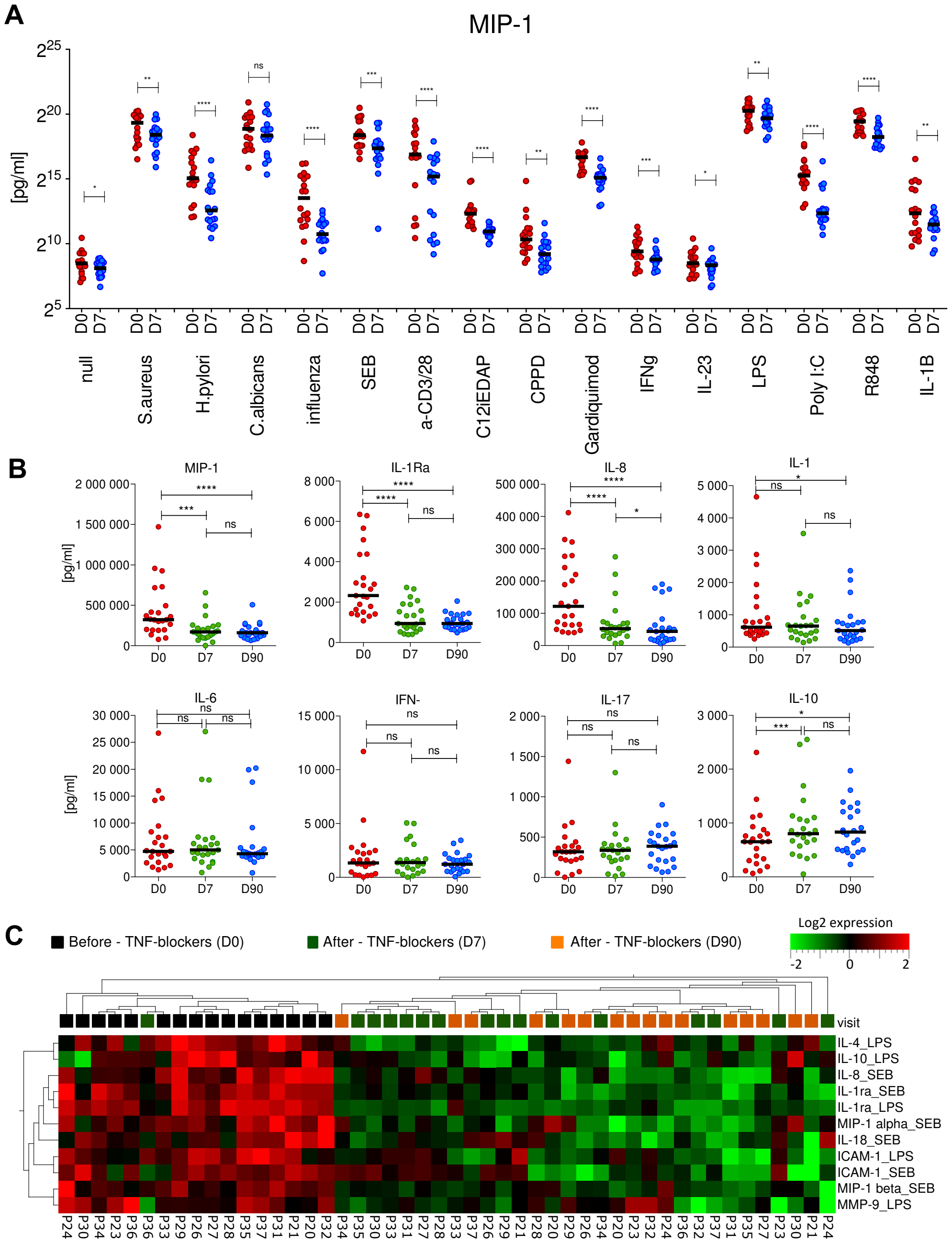

Supplementary Figure 2. Effects of different stimuli on protein signatures before and at different time points after anti-TNF treatment.

(**A**) Quantification of MIP-1β in TruCulture assay supernatants from 17 patients, at D0 (red) and D7 days (blue). The stimuli present in the TruCulture assays are indicated below the x-axis. (**B**) Quantification of proteins in supernatants of TruCulture assays stimulated with SEB from patients at D0, D7 and D90 after initiation of anti-TNF therapy. Horizontal bars indicate the median. Significance was determined using a Wilcoxon matched-pairs test (SpA patients before *versus* after treatment) and *P*-values are indicated above the graph (*: *P*<0.05; **: *P*<0.01; ***: *P*<0.001; ****: P<0.0001; ns: not significant). (**C**) The levels of 31 secreted molecules in response to LPS and SEB were compared in samples from 17 patients at D0 (black rectangles), D7 (green rectangles) and D90 (orange rectangles). The heatmap shows the levels of differentially secreted proteins (paired t-test, FDR ≤ 0.01, red indicates higher and green lower levels of protein secretion).

Supplementary Figure 3

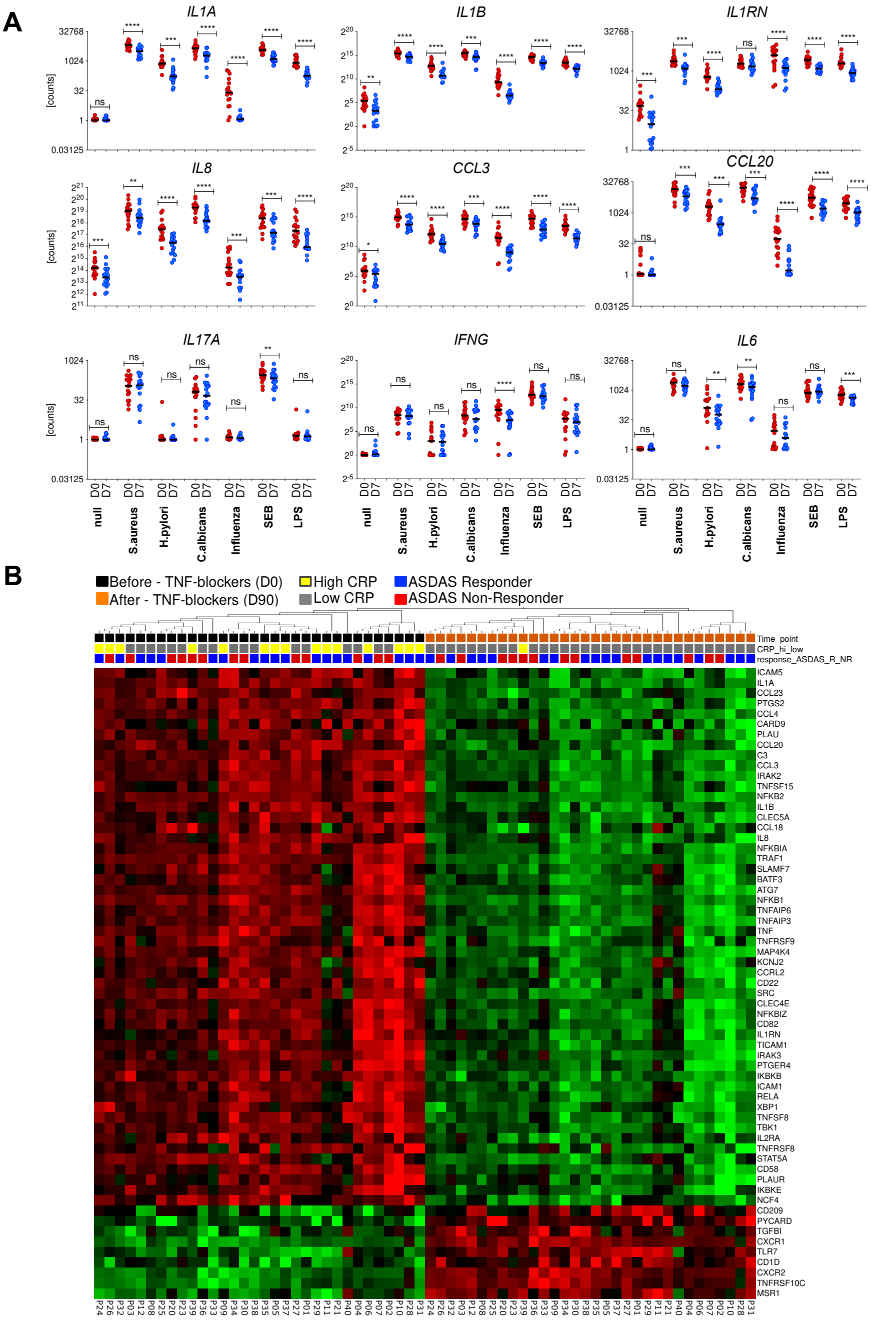

Supplementary Figure 3. TNF-blockers strongly affect key regulators of innate immune responses.
(**A**) Plots indicate expression level of genes encoding molecules with pro-inflammatory properties and of *IL17A, IFNG* and *IL6* for the unstimulated condition and 6 representative stimuli, in samples before (D0, red) and 7 days after (D7, blue) initiation of anti-TNF therapy. Stimuli present in the TruCulture assays are indicated below the x-axis (n = 17, FDR ≤ 0.05, as in Fig. 2). (**B**) Heatmap of differentially expressed genes, comparing samples from 32 patients before (D0, black rectangles) and 90 days (D90, orange rectangles) after initiation of anti-TNF therapy. Patients with CRP-levels > 6 mg/l are marked with yellow rectangles, while CRP-levels < 6 mg/l are indicated with grey rectangles. Patients responding to anti-TNF therapy (delta ASDAS ≥ 1.1) are marked in blue and non-responders (delta ASDAS < 1.1) are marked in red. A paired t-test with false-discovery rate FDR ≤ 0.01 and a fold-change threshold of ≥ 2 identified 61 genes (ranked by decreasing fold-change). Red indicates high-level, and green low level of gene expression, respectively. Data are normalized and log2 transformed.

Supplementary Figure 4

A

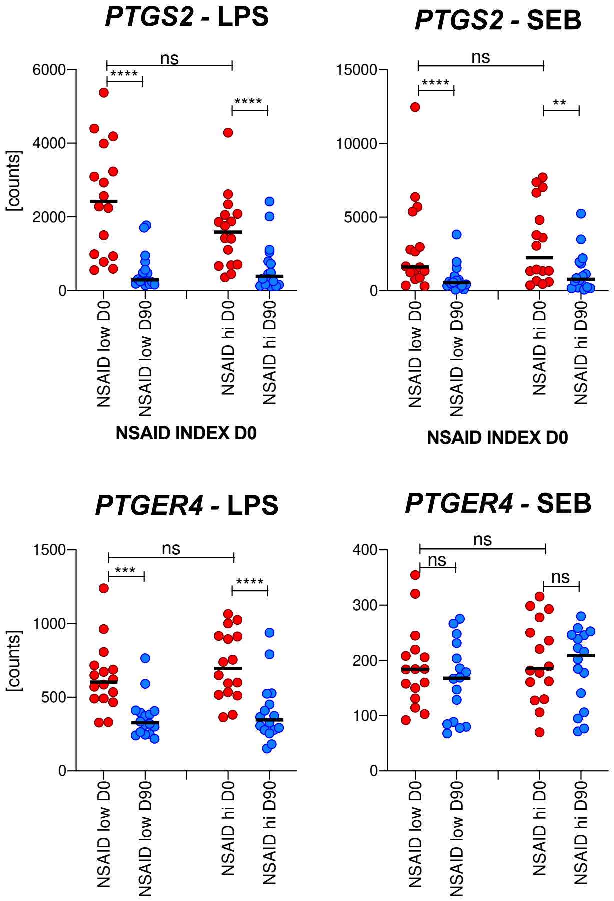

B

Patient_id	NSAID_D0	NSAID_D0_high/low
P01	11	low
P02	0	low
P03	150	hi
P04	84	hi
P05	177	hi
P06	89	hi
P07	100	hi
P08	46	low
P09	50	low
P10	50	low
P11	38	low
P12	50	low
P20	100	hi
P21	150	hi
P23	33,33	low
P24	0	low
P25	14,5	low
P26	83,33	hi
P27	50	low
P28	29	low
P29	100	hi
P30	11,11	low
P31	100	hi
P32	83,88	hi
P33	66,67	hi
P34	57	low
P35	100	hi
P36	6,33	low
P37	4,93	low
P38	100	hi
P39	110	hi
P40	76,34	hi

Supplementary Figure 4. The NSAID index was determined at baseline for the 32 patients for which gene expression data were available before (D0) and after (D90) TNFi treatment, and stratified patients according to the NSAID index (cut-off, median, **B**). *PTGS2* and *PTGER4* expression levels at D0 and D90 were plotted for the two groups of patients. Horizontal bars represent the median, and *P*-values are indicated above the graph (**: *P*<0.01; ***: *P*<0.001; ****: P<0.0001; ns: not significant).

Supplementary Figure 5

A

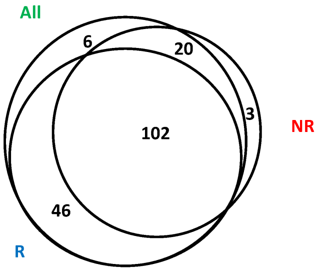

Supplementary figure 5. A. Gene expression data were analyzed in LPS-stimulated Truculture samples from 32 patients. Limma analysis was performed to compare gene expression at D0 versus D90 in 32 patients (all), or selectively in patients classified as Responders (R) or Non-responders (NR), according to ASDAS criteria. The Venn diagram shows the distribution of genes differentially expressed (adjusted p-value <0.05) in the indicated patient populations. The large majority (102) of differentially expressed genes was shared by all patient populations. Analysis of differentially expressed genes in NR patients alone identified 3 genes with significant changes between D0 and D90 specifically in these patients (*CD274*, *GBP1*, and *IL12B*).

B

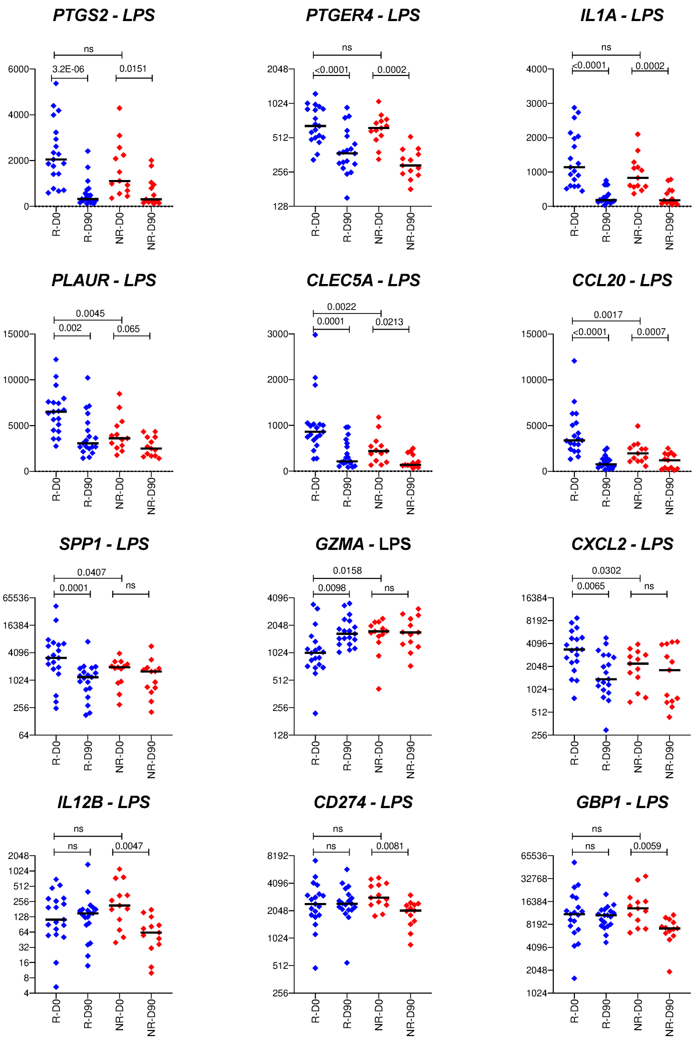

B. Selected genes from **A**. See also Supplementary Table 6.

Supplementary Figure 6

A

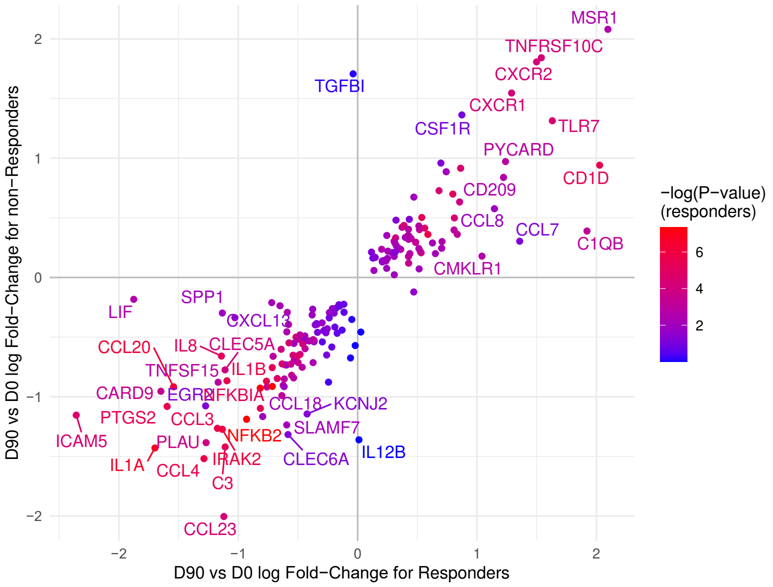

B

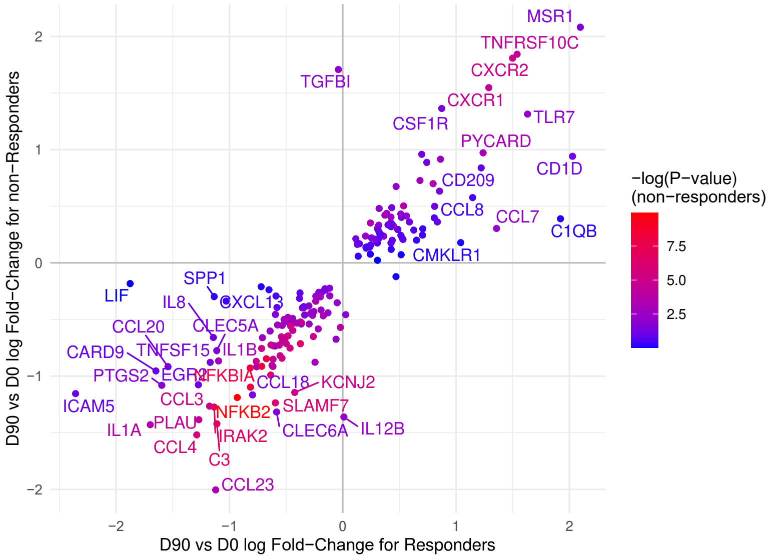

Supplementary figure 6. Differential gene expression between D0 (before treatment initiation) and D90 after treatment initiation.

Differential gene expression before and 90 days after TNFi treatment was calculated for Responders and Non-Responders (adjusted p-value <0.01, see **online supplementary table 6**), and Fold-changes of the differentially expressed genes were plotted for both populations. The labels identify the genes with log Fold-Change > 1 or < 1. The colors indicate the value of the adjusted p-value for each gene in Responders (**A**) and Non-responders (**B**)

Supplementary Figure 7

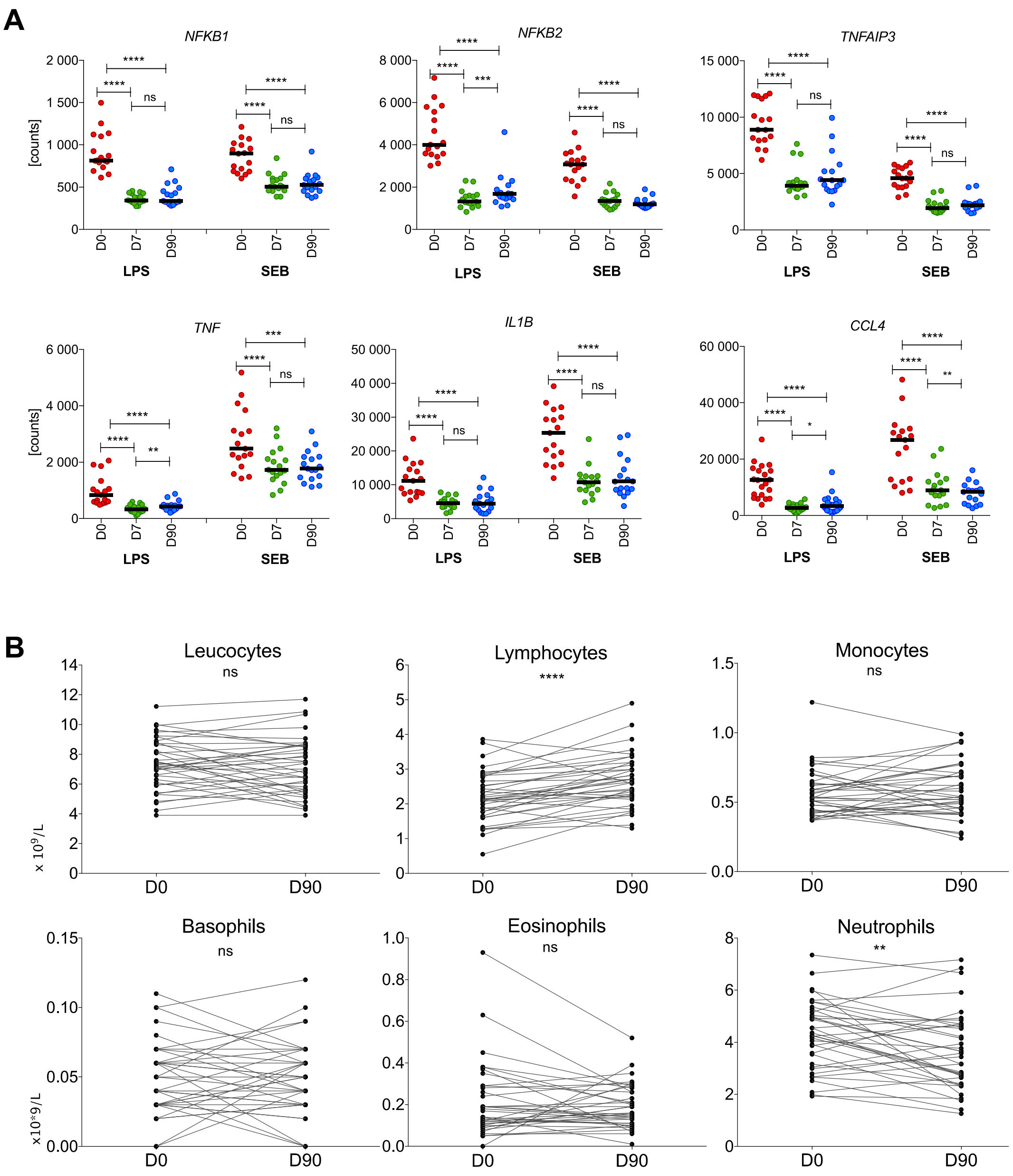

Supplementary Figure 7. The effects of TNF-blockers on immune responses can be detected after a single injection and remain stable over time.
(**A**) Plots indicate gene expression levels of immune genes from stimulation cultures containing LPS or SEB performed before (D0, in red), 7 days (D7, in green) and 90 days (D90, in blue) after initiation of anti-TNF therapy (17 patients). (**B**) Complete blood cell counts (Coulter counter) in 37 axSpA patients at D0 and D90 after initiation of anti-TNF therapy. Significance was determined using a Wilcoxon matched-pair test (values before *versus* after treatment). *P*-values are indicated above the graph (*: P<0.05; **: P<0.01; ***: P<0.001; ****: P<0.0001; ns: not significant). We noted a modest decrease (1.23-fold) of neutrophil counts and a 1.24-fold increase of lymphocyte counts after TNF therapy.

Supplementary Figure 8

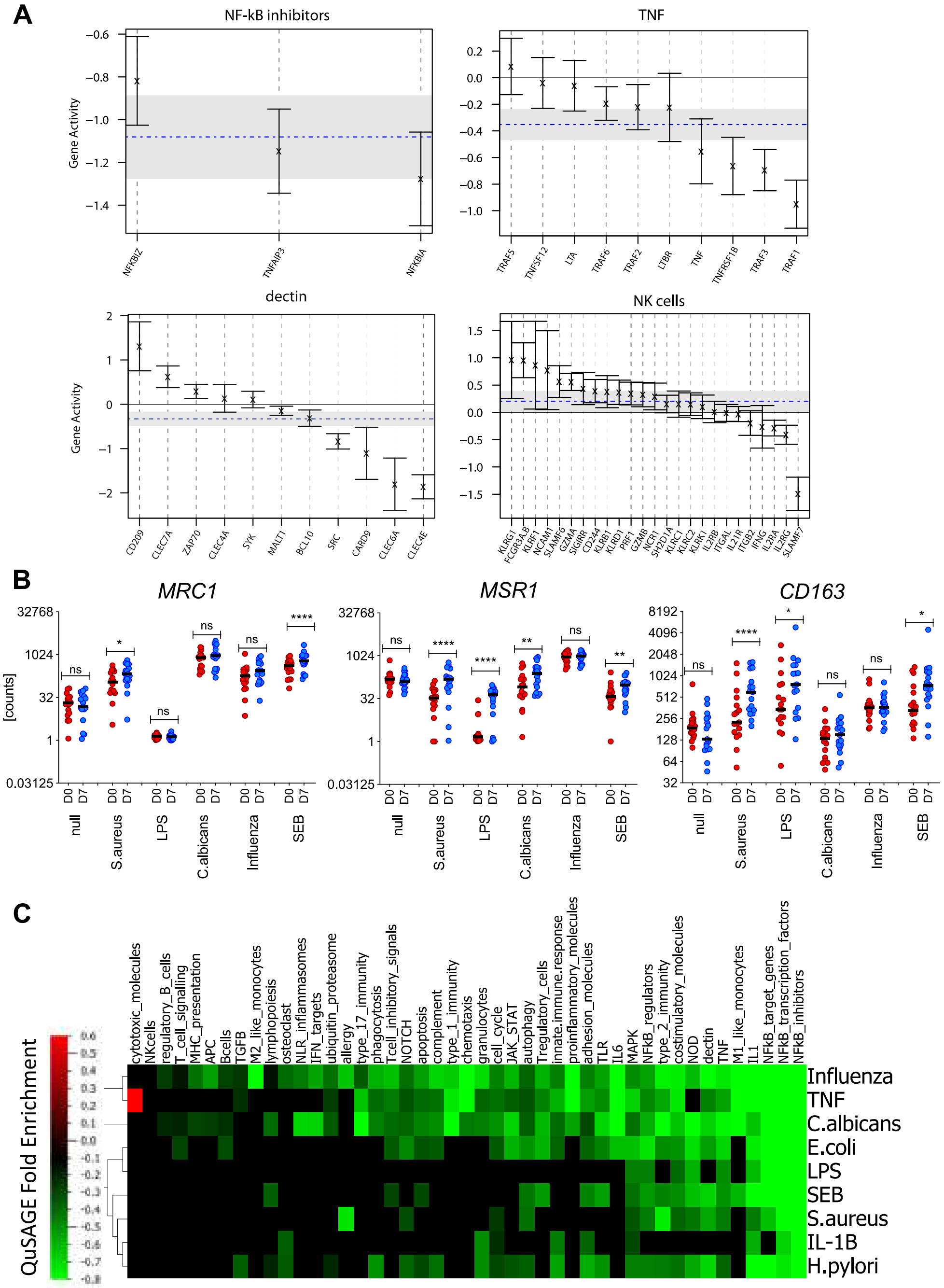

Supplementary Figure 8. Modular transcriptional framework to assess the signaling pathways affected by TNF-blockers in stimulated immune cells.

(**A**) Fold changes in gene activity in modules before and 7 days after initiation of anti-TNF therapy (D7 *versus* D0) for SEB stimulated samples. Represented are the mean fold-change and 95% confidence interval for individual genes in each module. Gene activity = 0 signifies no change. The horizontal dashed blue line and the grey band indicate the mean differential expression of genes in the module at D7, compared to D0, and the 95% confidence interval, respectively. (**B**) Plots indicate expression levels of M2-like monocyte-related genes for the null and 5 representative stimuli in Truculture assays from 17 patients before (D0, in red) and 7 days (D7, in blue) after initiation of anti-TNF therapy. (**C**) Heatmap representing QuSAGE fold-enrichment of gene sets in 9 different stimulated cultures from 12 SpA patients, at D90 after initiation of anti-TNF therapy *versus* D0. For each module, the mean fold-change is represented and color-coded to indicate increased (red) or decreased (green) module activity.

Supplementary Figure 9

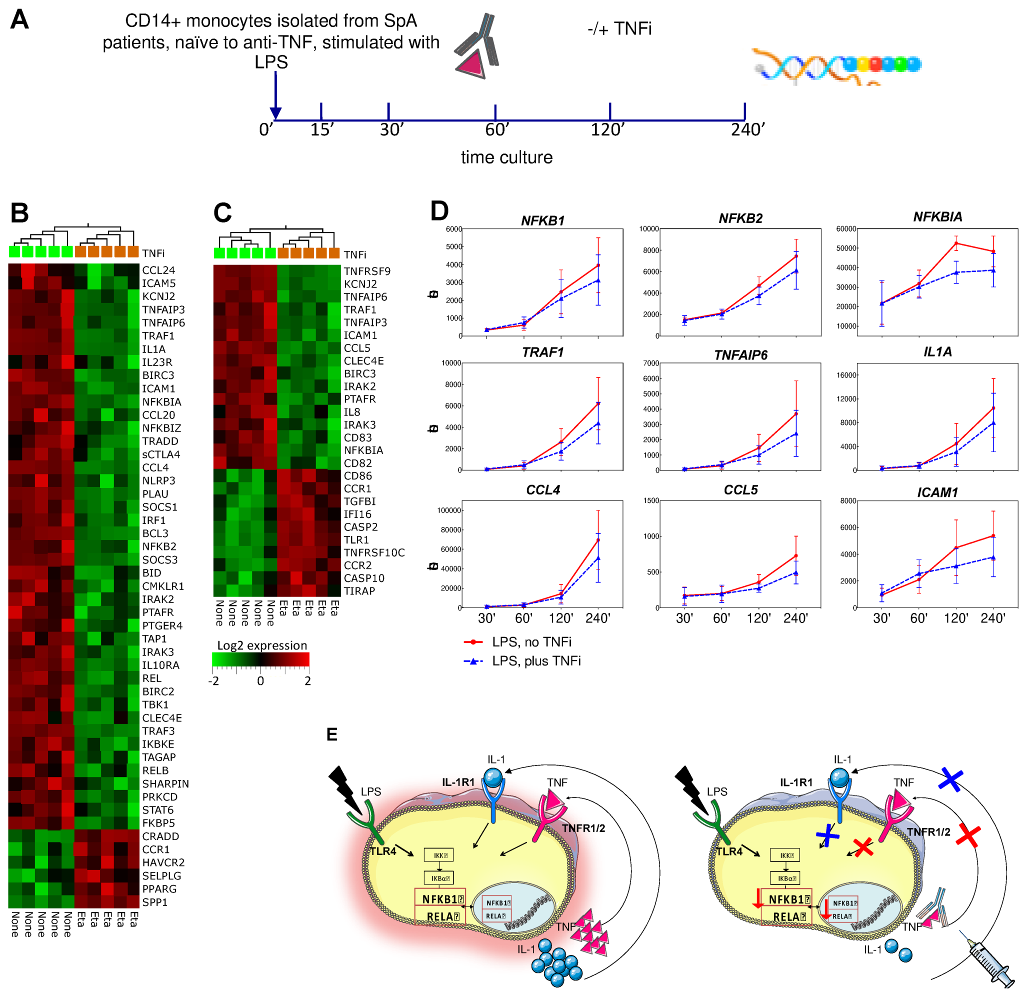

Supplementary Figure 9. TNF blockers break a TNF- and IL-1-dependent feed-forward loop of NF-κB activation in monocytes isolated from SpA patients

(**A**) Monocytes were isolated from 5 SpA patients and pre-incubated with or without TNFi (etanercept) for 10 minutes, prior to stimulation with LPS (20 ng/mL) for the indicated times. Gene expression was analyzed with the nCounter Human Immunology v2 panel. (**B, C**) Heatmaps show the top differentially expressed genes in monocytes in response to *in vitro* TNFi treatment after stimulation with LPS for 120 minutes (**B**) or 240 minutes (**C**). Orange and green rectangles distinguish samples pre-treated or not with TNFi, respectively. Gene expression analysis at the individual time points was performed using the Limma package with an adjusted *P*-value threshold of 0.1. (**D**) Expression kinetics of NF-kB target genes in LPS-stimulated monocytes cultured for the indicated times (minutes, horizontal axis). Monocytes were incubated with LPS (20 ng/mL) alone (red solid line), or pre-treated with TNFi for 10 minutes, followed by addition of LPS (blue dashed line). Shown are mean and standard deviation of 5 independent experiments. (**E**) Model for the intracellular mechanism of action of TNF-blockers.

Gene expression profiles of monocytes treated or not with Eta were strikingly different after 2 and 4 hours of LPS stimulation. A large proportion of the genes downregulated by TNFi at these time points were direct NF-kB target genes, such *NFKBIA*, *TNFAIP3*, *TNFAIP6*, or *IL1A*. The expression of NF-kB target genes in monocytes pre-treated with TNFi overlapped with untreated cultures during the first hour of stimulation, but diverged after 2 and 4 hours, compatibly with a positive feed-forward mechanism mediated by LPS-stimulated TNF production, which induces sustained activation of NF-kB and expression of its target genes, such as *IL1A* and *IL1B*, amplifying the inflammatory response.[13] Our data suggest that TNFi act by breaking the TNF- and the IL-1-dependent autocrine loops, dampening the activity of the NF-kB transcriptional cascade. Very similar results were obtained with monocytes isolated from 4 healthy donors, indicating that the action of TNFi on the NF-kB pathway is not dependent on the disease process (data not shown).

Supplementary Figure 10

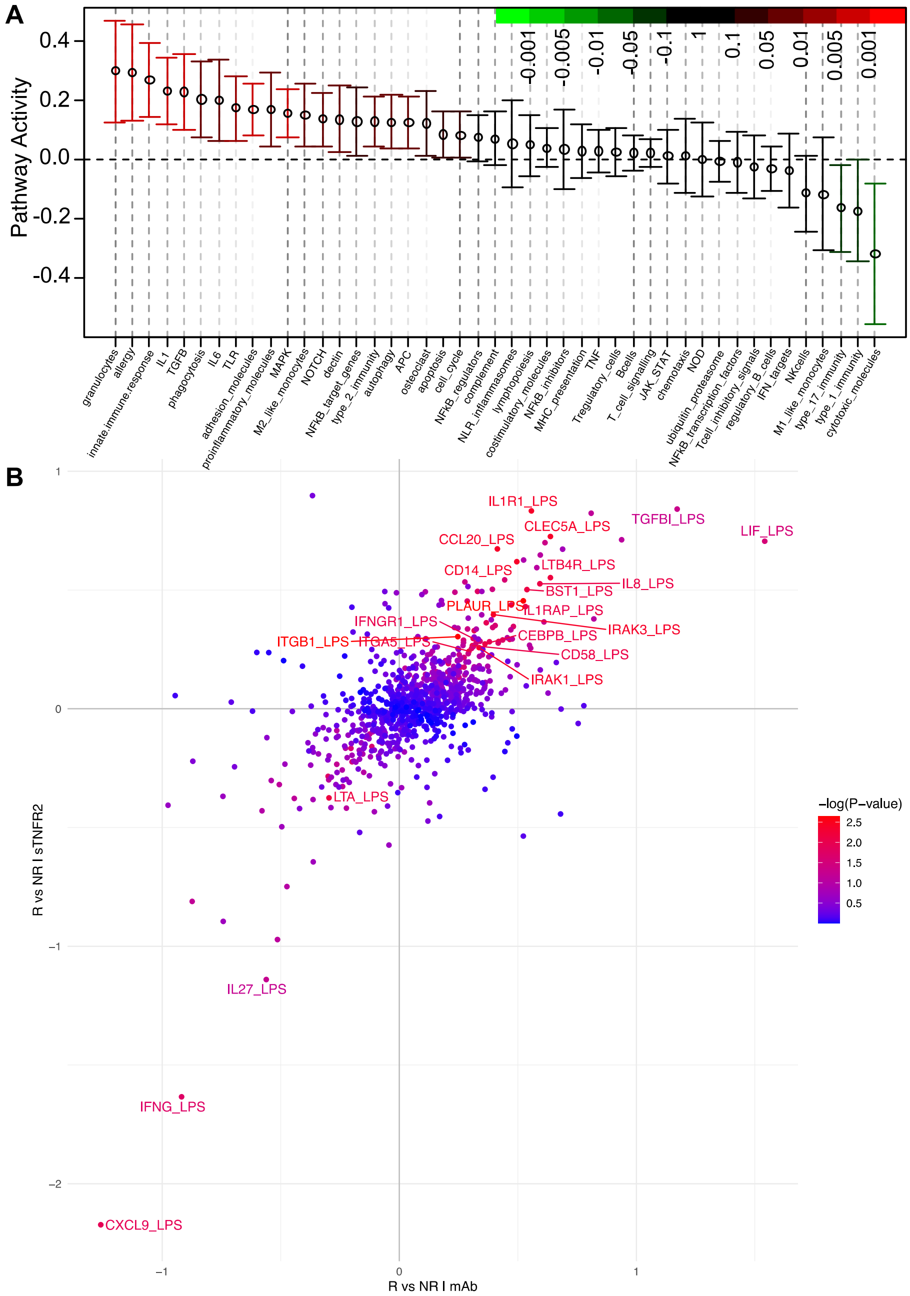

Supplementary Figure 10. A. Modular transcriptional repertoire analysis reveals differential activity of signaling pathways in responders versus non-responders before treatment initiation (D0).

Differential activity of 45 gene modules (**online supplementary table 5**) generated from 456 immune-related genes (80 patients). Whole-blood cultures were stimulated with LPS. For each gene module, the mean activity fold-change and 95% confidence interval are plotted and color-coded according to their FDR-corrected P-values (means compared to fold-change zero). Confidence intervals overlapping the horizontal dotted line indicate statistically significant increased or decreased module activity comparing responders and non-responders. **B.** Patients were grouped based on the type of treatment (etanercept (sTNFR2) versus monoclonal antibodies (mAb), see **online supplementary table 1**) and differential gene expression between responders and non-responders was calculated for each group at D0 (adjusted p-value <0.05, **table 2**), and fold-changes of the differentially expressed genes were plotted for both groups. The labels identify the genes with differential expression at adj. p-value < 0.05.

Supplementary Table 1. Demographic and clinical characteristics and response to anti-TNF treatment of the 80 axSpA patients included in the study

Patient ID	Gender	Age	CRP M0	ASDAS M0	CRP M3	ASDAS M3	Response ASDAS	Smoke	B27	Psoriasis	Uveitis	IBD	Anti-TNF
1	M	31	5.40	1.99	2.00	0.98	NR	1	1	0	0	0	Eta
2	F	37	2.10	3.71	2.00	3.39	NR	1	1	0	1	0	Ada
3	M	19	2.00	2.28	2.00	2.08	NR	0	0	0	0	0	Eta
4	M	37	2.00	1.96	2.00	0.97	NR	1	1	1	0	0	Eta
5	M	24	47.00	4.79	2.00	1.27	R	1	1	0	0	0	Eta
6	M	53	7.00	2.64	2.00	1.26	PR	1	1	0	1	0	Eta
7	M	54	2.00	1.13	5.00	1.71	NR	0	1	0	0	0	Eta
8	M	58	5.00	2.50	2.00	1.23	PR	0	1	0	1	0	Eta
9	M	34	17.00	4.39	2.00	1.58	R	0	1	0	0	0	Eta
10	M	42	9.00	3.03	2.00	0.94	R	1	0	1	0	0	Eta
11	M	23	51.00	4.46	2.00	0.87	R	1	1	0	0	0	Eta
12	F	42	2.00	2.16	2.00	0.83	PR	1	1	0	0	0	Eta
13	M	26	0.09	3.87	1.20	2.61	PR	0	1	0	0	0	Eta
14	M	26	2.48	1.28	0.00	0.87	NR	0	0	0	0	0	Eta
15	F	40	10.73	3.35	0.00	1.10	R	0	1	0	1	0	Eta
16	M	24	1.72	2.35	0.00	1.09	PR	0	1	0	0	0	Eta
17	F	27	11.35	2.58	2.00	0.64	PR	0	1	0	0	0	Eta
18	M	47	5.41	3.49	4.10	1.69	PR	0	1	1	1	0	Eta
19	M	30	1.11	3.53	0.50	2.58	NR	1	0	0	0	0	Eta
20	F	39	1.23	2.96	0.00	2.09	NR	1	1	1	0	0	Eta
21	F	21	20.15	2.35	2.80	1.08	PR	0	1	0	0	0	Eta
23	M	20	0.53	2.02	4.00	1.38	NR	0	1	0	1	0	Eta
24	F	58	37.50	4.75	7.00	2.97	PR	1	0	0	0	0	Eta
25	M	36	0.28	1.72	0.30	0.64	PR	0	1	0	0	0	Eta
26	M	48	7.46	1.61	0.00	0.77	NR	0	0	0	0	0	Eta
27	M	33	1.24	2.30	1.30	3.29	NR	0	1	1	1	0	Ada
28	F	40	21.44	4.68	0.00	0.87	R	1	1	1	0	0	Eta
29	M	50	27.45	4.39	3.10	2.29	R	1	1	0	1	0	Gol
30	M	57	2.63	3.07	1.20	2.67	NR	1	1	0	1	0	Gol
31	M	51	33.06	4.73	0.00	3.01	PR	1	1	0	1	0	Gol
32	M	58	16.97	3.25	0.00	1.24	R	0	0	1	0	0	Eta
33	F	24	5.72	2.78	4.00	1.16	PR	0	0	0	0	0	Eta
34	M	56	3.27	3.56	0.00	2.93	NR	0	1	0	1	0	Eta
35	F	38	39.38	4.43	2.00	0.75	R	0	1	0	1	1	Ada
36	F	47	0.68	2.50	0.50	1.48	NR	0	0	0	0	0	Eta
37	M	37	14.27	3.74	0.40	2.15	PR	0	1	0	1	0	Gol
38	M	43	4.09	3.10	2.00	1.71	PR	1	1	1	0	0	Eta
39	F	34	8.31	2.30	6.40	2.27	NR	0	1	0	0	0	Eta
40	M	43	1.11	2.32	3.00	0.92	PR	0	1	0	0	0	Eta
41	M	41	6.39	2.43	2.00	1.13	PR	0	1	1	0	0	Eta
42	M	55	15.24	2.88	21.10	2.63	NR	0	1	0	1	0	Eta
43	M	43	27.20	3.63	1.10	1.91	PR	0	1	0	1	0	Ada
44	M	47	0.18	2.14	0.50	1.63	NR	1	1	0	0	0	Eta
45	M	24	0.50	2.62	0.00	2.76	NR	1	1	0	0	0	Gol
46	M	27	0.35	3.50	0.00	1.33	R	1	1	1	0	0	Gol
47	M	44	4.18	3.21	2.70	2.08	PR	1	0	0	0	0	Eta
48	M	27	0.82	2.55	0.90	1.70	NR	1	1	0	0	0	Eta
49	F	52	15.20	4.07	9.00	1.87	R	0	0	0	1	1	Ada
50	F	27	3.58	3.09	4.00	2.82	NR	0	1	0	0	0	Gol
51	F	32	1.87	3.69	0.00	3.34	NR	1	0	0	0	1	Ada
52	M	27	2.00	2.56	1.00	0.96	PR	0	1	0	1	0	Eta
53	M	42	0.82	2.12	0.00	1.56	NR	1	1	0	0	0	Eta
54	M	45	3.39	3.16	1.00	1.78	PR	1	0	0	0	0	Gol

Patient ID	Gender	Age	CRP M0	ASDAS M0	CRP M3	ASDAS M3	Response ASDAS	Smoke	B27	Psoriasis	Uveitis	IBD	Anti-TNF
55	M	58	16.65	3.45	7.00	2.47	NR	1	0	0	0	0	Eta
56	M	41	10.74	2.80	0.00	0.71	R	0	1	0	1	0	Gol
57	M	39	3.35	3.29	1.90	1.89	PR	1	1	0	0	0	Gol
58	M	46	21.24	3.96	1.00	1.06	R	1	0	1	1	0	Ada
59	M	27	12.21	2.17	3.40	0.86	PR	1	1	0	0	0	Gol
60	F	29	16.38	2.56	1.00	0.94	PR	1	1	0	0	0	Gol
61	M	20	17.48	3.99	0.80	0.84	R	0	1	0	0	0	Eta
62	F	23	2.55	1.88	0.00	1.15	NR	1	1	0	0	0	Eta
63	M	32	0.48	1.92	2.00	0.64	PR	0	1	1	1	0	Eta
64	M	43	10.64	4.12	2.70	1.92	R	1	1	0	0	0	Eta
65	M	39	17.70	3.13	0.30	1.27	PR	0	1	0	1	0	Ada
66	F	64	15.23	3.88	6.00	1.72	R	0	1	0	1	0	Eta
67	M	57	12.53	4.19	2.10	3.45	NR	1	1	1	0	0	Eta
68	M	22	26.76	3.87	1.90	1.13	R	1	1	0	0	0	Eta
69	M	36	9.70	3.55	1.30	1.59	R	0	1	0	1	0	Eta
70	F	31	1.40	2.70	1.00	2.21	NR	1	1	0	0	0	Eta
71	F	21	62.00	4.61	51.80	3.12	R	1	1	0	0	0	Eta
72	F	55	1.00	2.75	1.00	0.94	R	1	0	0	0	0	Eta
73	M	57	28.60	3.90	2.00	0.94	R	0	1	0	1	0	Ada
74	F	48	1.00	2.69	1.00	1.62	NR	1	1	1	1	0	Ada
75	F	33	1.00	2.61	1.00	2.73	NR	0	0	0	0	0	Ada
76	F	53	7.80	2.64	1.40	1.41	R	0	1	1	1	0	Ada
77	F	25	6.40	2.10	5.00	1.06	NR	1	1	0	0	0	Eta
78	M	30	1.90	1.54	0.60	0.64	NR	0	1	0	0	0	Eta
79	M	31	19.20	3.60	2.00	2.40	R	1	1	0	0	0	Inf
80	M	23	20.00	3.30	2.00	0.90	R	0	1	0	0	0	Gol
81	M	26	8.20	3.10	2.00	1.10	R	1	1	1	0	0	Ada

Abbreviations are as follows: NR, ASDAS Non-Responder; PR, ASDAS Partial-Responder; R, ASDAS Responder; Eta, Etanercept; Ada, Adalimumab; Gol, Golimumab; Inf, Infliximab.

Supplementary Table 2. Drug dosage and anti-drug antibodies

Patient ID	anti-TNF	response ASDAS	Drug dosage (µg/ml)	Dosage ADAb (ng/ml)
P01	Etanercept	NR	2.8	<10
P02	Adalimumab	NR	2	<10
P03	Etanercept	NR	1.6	<10
P04	Etanercept	NR	1.2	<10
P05	Etanercept	R	<0.2	<10
P06	Etanercept	R	1.2	<10
P07	Etanercept	NR	3.2	<10
P08	Etanercept	R	1.6	<10
P09	Etanercept	R	2.8	<10
P10	Etanercept	R	2.8	<10
P11	Etanercept	R	<0.2	<10
P12	Etanercept	R	1.6	<10
P13	Etanercept	R	2.3	<10
P14	Etanercept	NR	0.7	<10
P15	Etanercept	R	2.6	<10
P16	Etanercept	R	3.1	<10
P18	Etanercept	R	0.3	<10
P19	Etanercept	NR	3.7	<10
P21	Etanercept	R	0.7	<10
P23	Etanercept	NR	2.2	<10
P24	Etanercept	R	1.3	<10
P25	Etanercept	R	1.9	<10
P26	Etanercept	NR	2.5	<10
P27	Adalimumab	NR	8.7	<10
P28	Etanercept	R	2.3	<10
P29	Golimumab	R	4.2	<2,5
P30	Golimumab	NR	3.6	<2,5
P31	Golimumab	R	2.7	<2,5
P32	Etanercept	R	0.9	<10
P33	Etanercept	R	2.6	<10
P34	Etanercept	NR	4.2	<10
P35	Adalimumab	R	>20	<10
P36	Etanercept	NR	3.2	<10
P37	Golimumab	R	1	<2,5
P39	Etanercept	NR	3.8	<10
P40	Etanercept	R	1.7	<10
P41	Etanercept	R	3.3	<10
P42	Etanercept	NR	2.8	<10
P44	Etanercept	NR	1.6	<10
P45	Golimumab	NR	2	<2,5
P46	Golimumab	R	1.8	<2,5
P47	Etanercept	R	>5	<10

Patient ID	anti-TNF	response ASDAS	Drug dosage (µg/ml)	Dosage ADAb (ng/ml)
P48	Etanercept	NR	2.3	<10
P49	Adalimumab	R	10.3	<10
50	Golimumab	NR	2	<2,5
51	Adalimumab	NR	10.2	<10
52	Etanercept	R	>5	<10
53	Etanercept	NR	2.1	<10
54	Golimumab	R	1.9	<2,5
55	Etanercept	NR	1.1	<10
56	Golimumab	R	1.8	<2,5
57	Golimumab	R	0.9	<2,5
58	Adalimumab	R	13.8	<10
59	Golimumab	R	<0,1	<2,5
60	Golimumab	R	4.8	<2,5
61	Etanercept	R	>5	<10
62	Etanercept	NR	1.8	<10
63	Etanercept	R	>5	<10
64	Etanercept	R	2.3	<10
65	Adalimumab	R	8.4	<10
66	Etanercept	R	3.3	<10
67	Etanercept	NR	<0,2	<10
68	Etanercept	R	>5	<10

Supplementary Table 3. Innate and Adaptive Immune Stimuli included in TruCulture Assays

**Stimulus**	**Concentration**	**Supplier**	**Sensor or Receptor**
Null		NA	
C12-iE-DAP	4 µg / ml	Invivogen	NOD1
α-CD3 + α-CD28	0.4µg/ml + 0.33 µg/ml		TCR
CPPD	100 µg/ml	Invivogen	NLRP3 & TLR2
Gardiquimod	3 µM	Invivogen	TLR7
HK *C. albicans*	10^7 bacteria	Invivogen	complex
HK *E.coli 0111:B4*	10^7 bacteria	Invivogen	complex
HK *H. pylori*	10^7 bacteria	Invivogen	complex
HK *S. aureus*	10^7 bacteria	Invivogen	complex
IFNγ (Imukin)	1000 IU/mL	Boehringer Ingelheim	IFNγR
IL-1β	25 ng/ml	Peprotec	IL1R
IL-1β + TNFα	25 ng/ml + 10 ng/ml		IL1R + TNFR
IL-23	50 ng/ml	Miltenyi Biotech	IL23R
Influenza (live)	1:700	Charles Rivers	Complex
LPS-EB (hi)	10 ng/ml		TLR4
BCG (Immucyst)	$3 * 10^5$ bacteria	Sanofi Pasteur	complex
poly I:C	20 µg/ml	Invivogen	TLR3
R848	1 µM	Invivogen	TLR7 & TLR8
Enterotoxin SEB	0.4 µg/ml	Bernhard Nocht Institute	TCR
TNFα	10 ng/ml	Miltenyi Biotech	TNFR
WGP	40 µg/ml	Invivogen	Dectin-1
Zymosan	300 µg/mL	Sigma-Aldrich	TLR2

Abbreviations are as follows: HK, heat killed; IU, international units. The stimulation conditions used for the preparation of TruCulture tubes are listed, with the indicated dose and commercial supplier.

Supplementary Table 4. Analytes measured in the supernatants of TruCulture Assays with Luminex xMAP technology

Analytes	Abbreviation	Units	LDD	LLOQ
Brain-Derived Neurotrophic Factor	BDNF	pg/mL	18.0	56.0
Eotaxin-1	Eotaxin-1	pg/mL	99.0	117.0
Factor VII	Factor VII	pg/mL	3000.0	2400.0
Granulocyte-Macrophage Colony-Stimulating Factor	GM-CSF	pg/mL	15.0	26.0
Intercellular Adhesion Molecule 1	ICAM-1	pg/mL	4200.0	6200.0
Interferon gamma	IFN-gamma	pg/mL	6.3	6.8
Interleukin-1 alpha	IL-1 alpha	pg/mL	0.8	1.1
Interleukin-1 beta	IL-1 beta	pg/mL	2.8	8.5
Interleukin-1 receptor antagonist	IL-1ra	pg/mL	38.0	59.0
Interleukin-2	IL-2	pg/mL	49.0	55.0
Interleukin-3	IL-3	pg/mL	8.3	8.6
Interleukin-4	IL-4	pg/mL	29.0	43.0
Interleukin-5	IL-5	pg/mL	3.5	6.0
Interleukin-6	IL-6	pg/mL	5.4	6.8
Interleukin-7	IL-7	pg/mL	30.0	41.0
Interleukin-8	IL-8	pg/mL	3.9	6.1
Interleukin-10	IL-10	pg/mL	4.9	8.1
Interleukin-12 Subunit p40	IL-12p40	pg/mL	220.0	450.0
Interleukin-12 Subunit p70	IL-12p70	pg/mL	25.0	37.0
Interleukin-15	IL-15	pg/mL	670.0	1200.0
Interleukin-17	IL-17	pg/mL	2.9	8.9
Interleukin-18	IL-18	pg/mL	31.0	42.0
Interleukin-23	IL-23	pg/mL	1300.0	3200.0
Macrophage Inflammatory Protein-1 alpha	MIP-1 alpha	pg/mL	43.0	48.0
Macrophage Inflammatory Protein-1 beta	MIP-1 beta	pg/mL	56.0	59.0
Matrix Metalloproteinase-3	MMP-3	pg/mL	55.0	70.0
Matrix Metalloproteinase-9	MMP-9	pg/mL	41000.0	33000.0
Monocyte Chemotactic Protein 1	MCP-1	pg/mL	107.0	83.0
Stem Cell Factor	SCF	pg/mL	97.0	222.0
Tumor Necrosis Factor alpha	TNF-alpha	pg/mL	16.0	24.0
Tumor Necrosis Factor beta	TNF-beta	pg/mL	39.0	58.0
Vascular Endothelial Growth Factor	VEGF	pg/mL	16.0	42.0

* The least detectable dose (LDD) was determined as the mean + 3 standard deviations of 200 blank readings. Results below the LDD are more variable than results above the LDD.

† The LLOQ (Lower Limit of Quantitation) is the lowest concentration of an analyte in a sample that can be reliably detected and at which the total error meets CLIA requirements for laboratory accuracy. As the LLOQ and the LDD values are independent from each other, on occasion the LLOQ is lower than the LDD.

Supplementary Table 5. Gene modules used in QuSAGE analysis

**Module**	**Genes**
Adhesion molecules	*APP, CD164, CD2, CD36, CD44, CD58, CD6, CD9, CD97, CD99, CEACAM1, CTNNB1, CX3CR1, DPP4, FN1, ICAM1, ICAM2, ICAM3, ICAM4, ICAM5, ITGA4, ITGA5, ITGA6, ITGAE, ITGAL, ITGAM, ITGAX, ITGB1, ITGB2, LGALS3, PECAM1, PLAU, PLAUR, PTK2, S100A9, SELE, SELL, SELPLG, SPP1, SRC, TGFBI, TNFAIP6*
Allergy	*CCL18, CCL5, FCER1A, IL13RA1, LTB4R, LTB4R2*
APC (Antigen Presenting Cells)	*BATF3, CCR7, CD14, CD163, CD1D, CD209, CD80, CD83, CD86, CD8A, CX3CR1, CXCR4, ITGAL, ITGAM, ITGAX, PDCD1LG2*
Apoptosis	*APP, BAX, BCAP31, BCL10, BCL2, BCL2L11, BID, CASP1, CASP10, CASP2, CASP3, CASP8, CD2, CD27, CD44, CDKN1A, CLEC5A, CRADD, CSF2RB, CTSC, CTSS, FAS, GZMB, LEF1, LGALS3, LTBR, MCL1, PDCD2, PRF1, PTK2, RAF1, TNFRSF10C, TNFRSF8, TNFSF10, TNFSF12, TNFSF15, TP53*
Autophagy	*ABL1, ATG10, ATG12, ATG16L1, ATG5, ATG7, IFI16, PTPN22, S100A8, S100A9, TOLLIP, XBP1*
B-cells	*BCL6, BLNK, BST1, BST2, BTK, CD19, CD22, CD24, CD27, CD79A, CD79B, CD80, CD81, CD99, CR2, CXCL13, ENTPD1, IFITM1, IL4R, IL6R, IRF8, ITGA5, LEF1, LILRB3, MS4A1, PAX5, PRDM1, PRKCD, PTPN6, SYK, TNFRSF13C, TNFRSF8, TNFSF13B, TNFSF8, ZAP70*
Cell cycle	*ABL1, AHR, BAX, BCL2, BID, CCND3, CDKN1A, IKZF1, MAPK1, PML, PRKCD, PTK2, RARRES3, S100A8, S100A9, SRC*
Chemotaxis	*CCL13, CCL18, CCL19, CCL2, CCL20, CCL22, CCL23, CCL24, CCL3, CCL4, CCL5, CCL7, CCL8, CCR1, CCR2, CCR5, CCR6, CCR7, CCRL2, CD99, CX3CR1, CXCL1, CXCL10, CXCL11, CXCL13, CXCL9, CXCR1, CXCR2, CXCR3, CXCR4, CXCR6, IL16, IL8, LGALS3, PPBP*
Complement	*C1QB, C1QBP, C2, C3, CASP1, CASP10, CASP3, CCL5, CD36, CD40LG, CD46, CD59, CEBPB, CFB, CFD, CFP, CR1, CR2, CTSC, CXCL1, FCER1G, FYN, ITGAM, ITGAX, ITGB2, LTF, PLAUR, PRKCD, PSMB9, RAF1, SERPING1, SRC, TNFAIP3*
Costimulatory molecules	*ADA, CD27, CD28, CD40, CD40LG, CD48, CD6, CD79B, CD80, CD82, CD86, CLEC5A, DPP4, ICOS, ICOSLG, MBP, PDCD1LG2, TAGAP, TNFRSF4, TNFRSF8, TNFRSF9, TNFSF12, TNFSF15, TNFSF4, TNFSF8, TRAF1*
Cytotoxic molecules	*GNLY, GZMA, GZMB, GZMK, IFNG, KLRD1, KLRF1, PRF1*
Dectin	*BCL10, CARD9, CD209, CLEC4A, CLEC4E, CLEC6A, CLEC7A, MALT1, SRC, SYK, ZAP70*
Granulocytes	*CCRL2, CD164, CD24, CD44, CLEC5A, CSF2, CSF3R, CXCL1, CXCR1, CXCR2, FCGR1A.B, FCGR3A.B, IL3, IL8, ITGAL, ITGAM, ITGAX, ITGB2, LTB4R, LTB4R2, LTF, MME, NCF4, SELL*
IFN targets	*BST2, CXCL10, IFI35, IFIH1, IFIT2, IFITM1, IFNA1.13, IFNAR1, IFNAR2, IRF1, IRF3, IRF4, IRF5, IRF7, IRF8, JAK1, MX1, PSMB8, TMEM173, TYK2*
IL1	*EGR1, IL18, IL18R1, IL18RAP, IL1A, IL1B, IL1R1, IL1R2, IL1RAP, IL1RL1, IL1RN, IRAK1, IRAK2, IRAK3, IRAK4, MYD88, SIGIRR, TOLLIP, TRAF6*
IL6	*IL6, IL6R, IL6ST*
Innate immune response	*ABL1, APP, BCL10, C1QBP, CD14, CLEC5A, CLEC7A, FCER1G, IKBKG, IL1RAP, IRAK1, IRAK4, LY96, NLRP3, S100A8, S100A9, TLR2, TLR4, TOLLIP*
JAK_STAT	*CISH, JAK1, JAK2, JAK3, PTPN2, PTPN6, PTPRC_all, SOCS1, SOCS3, STAT1, STAT2, STAT3, STAT4, STAT5A, STAT5B, STAT6, TYK2*
Lymphopoiesis	*CXCR4, IKZF1, IKZF2, IKZF3, NT5E, PAX5, RUNX1*
M1-like monocytes	*CCL19, CCL20, CCL5, CCL8, CCR7, CD80, CD86, CXCL10, CXCL11, CXCL9, IDO1, IFNGR1, IL12B, IL1R1, IL23A, IL2RA, MARCO, PTGS2, SOCS3*

Module	Genes
M2-like monocytes	*CCL13, CCL18, CCL2, CCL22, CCL24, CD163, CD209, CD36, CLEC7A, EGR2, FCER1A, FN1, IL10, IL1R2, IL1RAP, IL1RN, IL21R, IL4R, IRF4, MRC1, MSR1*
MAPK	*CD83, DUSP4, MAP4K1, MAP4K2, MAP4K4, MAPK1, MAPK14, MAPKAPK2, RAF1*
MHC presentation	*B2M, BCAP31, CD74, CTSS, HLA.A, HLA.B, HLA.C, HLA.DMA, HLA.DMB, HLA.DOB, HLA.DPA1, HLA.DPB1, KLRC1, KLRC2, KLRC3, KLRC4, KLRD1, KLRF1, KLRG1, KLRK1, LAMP3, LILRA1, LILRA2, LILRA3, LILRA6, LILRB1, LILRB2, LILRB4, MR1, MS4A1, NCF4, TAP1, TAP2, TAPBP, TNFSF4, XBP1*
NFkB inhibitors	*NFKBIA, NFKBIZ, TNFAIP3*
NFkB regulators	*BCL10, BTK, CHUK, IKBKAP, IKBKB, IKBKE, IKBKG, MALT1, MAP4K4, TBK1, TRAF4*
NFkB target genes	*BCL2, BCL3, CCL13, CCL19, CCL4, CXCL2, CYBB, ICAM1, IL1B, IL8, NFKBIA, PLAU, PTGS2, TNF, TNFAIP3, TNFSF13B, TRAF1, TRAF2*
NFkB transcription factors	*NFKB1, NFKB2, RELA, RELB*
NK-cells	*CD244, FCGR3A.B, GZMA, GZMB, IFNG, IL21R, IL2RA, IL2RB, IL2RG, ITGAL, ITGB2, KLRB1, KLRC1, KLRC2, KLRD1, KLRF1, KLRG1, KLRK1, NCAM1, NCR1, PRF1, SH2D1A, SIGIRR, SLAMF6, SLAMF7*
NLR_inflammasomes	*BCL2, CASP1, GBP5, NLRP3, PYCARD*
NOD	*CARD9, NOD1, NOD2, TRAF4, TRAF6*
NOTCH	*APP, IL2RA, NCR1, NFIL3, NOTCH1, NOTCH2, TGFB1, TGFBR2*
Osteoclast	*CEBPB, CSF1, CSF1R, CTNNB1, GPR183, LILRA1, LILRA2, LILRA3, LILRA5, LILRA6, MAPK14, NFATC1, SYK, TFRC, TRAF6*
Phagocytosis	*CYBB, ETS1, FCER1A, FCER1G, FCGR1A.B, FCGR2A, FCGR2A.C, FCGR2B, FCGR3A.B, FCGRT, ICAM3, ICAM5, IRF8, ITGAL, ITGAM, ITGAX, ITGB2, MARCO, PECAM1, SLAMF1*
Proinflammatory molecules	*CCL13, CCL18, CCL19, CCL2, CCL20, CCL22, CCL23, CCL24, CCL3, CCL4, CCL5, CCL7, CCL8, CCR1, CCR2, CCR5, CD163, CMKLR1, CSF1, CSF1R, CSF2, CXCL1, CXCL2, CXCR1, CXCR2, CXCR4, IL1B, IL32, IL6, IL6R, IL6ST, IL8, LILRA5, LITAF, MIF, PTAFR, PTGER4, PTGS2, S100A8, S100A9, TNF*
Regulatory B-cells	*CD19, CD1D, CD24, CD27, CD40, CD5, CD80, CD86, ICOSLG, IL10, PAX5, TFRC, TGFB1, TNFRSF13C*
T-cell signaling	*CD247, CD28, CD3D, CD3E, CD4, CD45R0, CD45RA, CD45RB, CD7, CD8A, CD8B, FYN, IL2RA, IL2RB, IL2RG, LCK, LCP2, NFATC1, NFATC2, NFATC3, PTPN22, PTPRC_all, ZAP70*
T-cell inhibitory signals	*BTLA, CAMP, CD244, CD274, CD276, CD5, CD96, CTLA4_all, CTLA4.TM, HAVCR2, IDO1, LAG3, PDCD1LG2, sCTLA4, TIGIT, TNFRSF14*
TGFB	*MAPK1, SKI, SMAD3, SMAD5, TGFB1, TGFBI, TGFBR1, TGFBR2*
TLR	*BCL10, CD14, IRAK1, IRAK2, IRAK4, LY96, MALT1, MYD88, TBK1, TICAM1, TIRAP, TLR1, TLR2, TLR3, TLR4, TLR7, TLR8, TOLLIP*
TNF	*LTA, LTBR, TNF, TNFRSF1B, TNFSF12, TRAF1, TRAF2, TRAF3, TRAF5, TRAF6*
T-regulatory cells	*CTLA4_all, CTLA4.TM, EGR2, ENTPD1, FOXP3, IL10, IL2, IL2RA, IL2RB, IL2RG, LAG3, LGALS3, NT5E, RUNX1, sCTLA4, STAT5A, STAT5B, TGFB1*
Type 1 immunity	*BATF3, CSF2, CXCR3, EBI3, GZMB, IFNG, IFNGR1, IL12B, IL12RB1, IL27, PRF1, STAT1, STAT4, TBX21, TNF*
Type 17 immunity	*AHR, BATF, CCR6, IL12B, IL17A, IL17F, IL21, IL22, IL23A, IRF4, KLRB1, MAF, STAT3, ZBTB16*
Type 2 immunity	*CCL18, CEBPB, CXCR4, CXCR6, IL13, IL1RL1, IL4R, STAT6*
Ubiquitin / proteasome	*CUL9, PSMB10, PSMB5, PSMB7, PSMB8, PSMB9, PSMC2, PSMD7, UBE2L3*

Supplementary Table 6. Differential gene expression between D0 (before treatment initiation) and D90 after treatment initiation*

	ALL patients (n=32)		RESPONDERS n=(19)		NON RESPONDERS (n=13)	
**Gene ID**	**logFC**	**adj.P.Val**	**logFC**	**adj.P.Val**	**logFC**	**adj.P.Val**
**ABL1**	0.253176758	0.001442703	0.30586301	0.006636767	0.176173775	0.222400494
**ARHGDIB**	0.21558608	3.61186E-06	0.201240695	0.001991642	0.236552413	0.00577187
**ATG7**	-0.806553689	6.3366E-11	-0.76352244	4.30165E-06	-0.869445515	1.23085E-05
**B2M**	-0.186180517	0.003718453	-0.11403	0.232445	-0.291628825	0.004017264
**BATF**	-0.418270605	3.22532E-05	-0.25939167	0.086164035	-0.650478282	1.23085E-05
**BATF3**	-0.824332688	4.53682E-08	-0.76012543	0.000211741	-0.918174066	0.000377461
**BCL10**	-0.260307478	4.88768E-05	-0.23718	0.010894	-0.294103926	0.003440946
**BCL2L11**	0.310465201	1.08621E-05	0.361489029	0.001221817	0.235891913	0.016977221
**BID**	-0.386253923	0.003427715	-0.335769	0.07856512	-0.460039575	0.029254642
**BLNK**	0.642740956	0.000113894	0.83513124	0.000559507	0.361555156	0.088286622
**BTK**	0.407795145	1.10241E-06	0.485467354	4.54742E-05	0.294274224	0.025728486
**C1QB**	1.298878127	0.002256578	1.920997082	0.000728495	0.389627346	0.67250275
**C1QBP**	0.175416058	0.009720333	0.243298384	0.009080724	0.076203	0.597687
**C3**	-1.236195878	1.73288E-12	-1.10986335	4.86561E-06	-1.420835733	2.24648E-07
**CARD9**	-1.366533982	3.75512E-05	-1.64858428	0.000384184	-0.954306617	0.094026687
**CASP1**	-0.256446002	0.006838877	-0.13	0.354265	-0.441256469	0.002979257
**CASP2**	0.187295816	1.20777E-05	0.21786434	0.00065451	0.142619	0.070906
**CASP8**	0.153024118	0.00628384	0.14024682	0.09774861	0.171698632	0.060545374
**CCL18**	-0.945888265	0.0003865	-0.79553	0.010676	-1.16564	0.030486
**CCL20**	-1.287913287	4.62521E-08	-1.5420472	4.76266E-07	-0.916486796	0.03208227
**CCL22**	0.64122106	0.000282579	0.80783953	0.001002966	0.397701757	0.20070615
**CCL23**	-1.479951808	1.03024E-07	-1.12084465	4.69475E-05	-2.004800729	0.000650271
**CCL3**	-1.21153499	2.8261E-11	-1.17490667	3.20446E-06	-1.265068689	1.88176E-05
**CCL4**	-1.381514235	1.53306E-12	-1.28750358	1.79524E-06	-1.518914417	1.86125E-06
**CCL7**	1.397525731	0.006838877	1.357208422	0.067879816	0.303937794	0.005981057
**CCL8**	0.91478911	0.006054825	1.146072573	0.013102692	0.576759433	0.342077344
**CCND3**	0.30486017	5.86801E-07	0.305491269	0.000384184	0.303938	0.005981
**CCR2**	0.542001344	0.006267441	0.706916022	0.003047309	0.300972	0.496146
**CCR6**	0.373827331	0.00052979	0.399839161	0.009836448	0.33581	0.065575
**CCRL2**	-0.710506217	1.34062E-07	-0.61429394	0.001127077	-0.851124167	4.95602E-05
**CD19**	0.408952327	6.90133E-05	0.486926825	0.000446304	0.294989599	0.098863245
**CD1D**	1.58606564	1.25323E-07	2.027168669	3.20446E-06	0.941377	0.050676
**CD209**	1.066351628	0.00053409	1.221933753	0.002604643	0.838962368	0.145168481
**CD22**	-0.689190454	4.52499E-06	-0.70825783	0.00127915	-0.661322747	0.003015926
**CD274**	-0.169834846	0.229203138	0.027022	0.915602	-0.457548233	0.008113729
**CD3E**	0.142228304	0.008427814	0.127954284	0.127351976	0.16309	0.05572
**CD4**	0.175907644	0.007214366	0.207115	0.020234	0.130296392	0.315832453
**CD44**	-0.430761404	8.68282E-08	-0.36339167	0.000947654	-0.529224861	5.38549E-05
**CD48**	-0.15982423	0.006749758	-0.11564771	0.196980747	-0.22439	0.015094
**CD53**	-0.213420521	0.005641292	-0.18267	0.112217	-0.258361946	0.026996209
**CD58**	-0.510167608	4.77139E-08	-0.51696989	4.9506E-05	-0.500225805	0.000528138
**CD74**	0.255761012	0.000311337	0.288231669	0.013884888	0.208304	0.01278
**CD79A**	0.502219684	3.89983E-08	0.562565512	8.20946E-06	0.414021936	0.006403097
**CD79B**	0.470882264	1.88402E-05	0.518391159	0.000578627	0.401446188	0.041019776
**CD82**	-0.660170925	1.48723E-06	-0.61122447	0.002614556	-0.731708058	5.67222E-05
**CD83**	-0.436272449	9.93638E-05	-0.36562	0.024918	-0.53953624	0.00283801

	ALL patients (n=32)		RESPONDERS n=(19)		NON RESPONDERS (n=13)	
Gene ID	logFC	adj.P.Val	logFC	adj.P.Val	logFC	adj.P.Val
CD86	0.386163476	0.001303576	0.321470565	0.053924554	0.480714654	0.011091724
CDKN1A	-0.46189628	1.87844E-05	-0.44215002	0.004284074	-0.490756204	0.003520628
CFB	-0.50162	0.016864	-0.2442	0.489735	-0.877853933	0.001255433
CFP	-0.395356865	0.00853923	-0.29629492	0.226126483	-0.540139709	0.004705888
CLEC4E	-0.661224	1.60969E-08	-0.55272992	0.000372592	-0.819792266	2.88963E-05
CLEC5A	-0.974540425	8.55814E-06	-1.11103278	0.000132995	-0.775051593	0.021319142
CLEC6A	-0.88133631	0.001315286	-0.58318589	0.059775076	-1.317094621	0.018794245
CMKLR1	0.691054017	0.004847332	1.041467919	0.002100337	0.178911	0.712636
CR1	0.700674902	8.68282E-08	0.682214063	5.50216E-05	0.727656129	0.001976613
CSF1R	1.072382648	0.007543374	0.873350027	0.089829004	1.36327648	0.0376294
CSF3R	0.884218586	9.30584E-08	0.862728133	3.61882E-05	0.91562771	0.003520628
CTLA4.TM	0.30033701	0.002287422	0.322429	0.013816	0.268049	0.14634
CTSS	-0.291748424	0.00187452	-0.17162511	0.232253804	-0.467313265	0.000650271
CXCL13	-0.747634709	0.009915684	-1.02821	0.024732	-0.33756904	0.395990086
CXCL2	-0.512839732	0.012003273	-0.71951007	0.006462195	-0.21078	0.63822
CXCR1	1.393945583	1.35617E-09	1.289375477	5.13172E-05	1.546778815	3.05185E-05
CXCR2	1.624132901	4.78263E-10	1.498542502	4.9506E-05	1.8076881	1.01218E-05
CXCR3	0.455116599	0.003934521	0.432833349	0.062474661	0.487684	0.057755
EGR2	-1.193637301	0.005169237	-1.27371	0.051147	-1.07661219	0.078384248
FCGR3A.B	0.50565039	0.000473349	0.470280905	0.024887141	-0.12161	0.741475
GBP1	-0.30919	0.061949	-0.05915	0.856059	-0.674629045	0.00594838
GFI1	-0.331428841	0.000364426	-0.29799237	0.016222066	-0.380297523	0.01880011
GZMA	0.335012971	0.019117139	0.51511183	0.009836448	0.071792	0.797502
HLA.DMA	0.398497551	1.97889E-06	0.447338798	0.000804489	0.327114189	0.003135577
HLA.DMB	0.400221403	3.35841E-07	0.524745205	9.40411E-06	0.218225078	0.062579352
HLA.DPA1	0.399941187	2.11781E-05	0.531855391	0.0002424	0.207144	0.108904
HLA.DPB1	0.324440692	3.22532E-05	0.426154747	0.000442848	0.175781689	0.106433258
ICAM1	-0.585892693	3.82126E-09	-0.49916988	0.000283245	-0.712641417	1.23085E-05
ICAM5	-1.868511919	3.36776E-06	-2.35700327	2.63903E-05	-1.15456	0.097668
ICOSLG	-0.350217647	0.001818817	-0.28376	0.090759	-0.447349566	0.005241155
IFITM1	0.340797741	0.000117897	0.43726364	0.001416312	0.199809119	0.10777694
IFNA1.13	-0.482674487	0.008038195	-0.65052326	0.005755428	-0.23736	0.545243
IKBKAP	0.275415395	0.009915684	0.316681853	0.06165645	0.215102878	0.095971671
IKBKB	-0.586494261	4.05119E-10	-0.54132876	4.23135E-05	-0.652505384	2.07119E-05
IKBKE	-0.5041171	6.78676E-08	-0.4404755	0.000846567	-0.59713175	8.36705E-05
IKZF3	0.178708824	0.007872859	0.206137	0.020362	0.138621	0.260855
IL12B	-0.54674	0.08931	0.010747	0.984681	-1.361529376	0.004705888
IL12RB1	0.468047093	0.003956999	0.65134258	0.007385616	0.200153689	0.440823919
IL1A	-1.588912275	2.15941E-11	-1.69809729	2.14274E-07	-1.429334177	0.000314071
IL1B	-1.002162478	9.87292E-10	-1.0953298	1.79524E-06	-0.865994858	0.001269259
IL1RN	-0.651814707	2.97169E-05	-0.59560873	0.008921978	-0.733961901	0.002979257
IL21R	0.403332947	0.000582278	0.513279894	0.002100337	0.242641	0.227161
IL2RA	-0.541302821	8.09206E-05	-0.53361	0.013885	-0.552547674	0.001989851
IL2RG	-0.361122445	6.54302E-08	-0.33909897	0.00065451	-0.393310596	6.46097E-05
IL8	-0.945016376	1.10241E-06	-1.14106032	7.03263E-06	-0.658490605	0.06012345
IRAK2	-1.190504303	1.57408E-13	-1.13387517	7.19758E-07	-1.27326996	2.24648E-07
IRAK3	-0.62522298	2.44206E-06	-0.64086859	0.000155825	-0.602356317	0.005979485
IRF3	0.800892843	0.000443594	0.742095693	0.009345021	0.886827	0.05073

	ALL patients (n=32)		RESPONDERS n=(19)		NON RESPONDERS (n=13)	
**Gene ID**	**logFC**	**adj.P.Val**	**logFC**	**Gene ID**	**logFC**	**adj.P.Val**
**IRF5**	-0.365246966	0.001919254	-0.21617117	0.214886147	-0.583126974	0.001194602
**IRF8**	0.302224424	0.000790595	0.427388287	0.000278178	0.119292624	0.543391483
**ITGA4**	0.197001693	0.002680241	0.237105427	0.009836448	0.138389	0.273789
**ITGA6**	0.479899036	1.01785E-05	0.512806788	0.001481542	0.431803091	0.011464671
**KCNJ2**	-0.716060168	7.33496E-06	-0.42315	0.054942	-1.144152681	1.23085E-05
**KLRB1**	0.383235294	0.000150528	0.426704773	0.001982921	0.319703	0.078384
**LCK**	0.155076478	0.002051829	0.115774681	0.077399912	0.212517566	0.043546384
**LGALS3**	-0.346184251	0.000426199	-0.26579	0.069423	-0.463680833	0.002189262
**LIF**	-1.188262593	0.010734849	-1.87595349	0.002606424	-0.18318	0.851711
**LILRA5**	0.764340852	3.09327E-06	0.854163228	0.000207665	0.633061994	0.02163132
**LITAF**	-0.282569407	0.003504842	-0.1912827	0.195135199	-0.415988437	0.002785657
**LY96**	0.394479046	1.01785E-05	0.422931162	0.000680084	0.352895	0.019343
**MAF**	0.803491056	0.002838368	0.697089022	0.057124202	0.959001721	0.047282386
**MAP4K1**	0.323772798	7.34957E-07	0.317921328	0.000139679	0.332324946	0.008199687
**MAP4K4**	-0.731686012	2.88379E-10	-0.71535371	6.31502E-06	-0.755556305	1.23085E-05
**MAPKAPK2**	-0.23123666	0.000507941	-0.21108877	0.030291873	-0.260683578	0.017436201
**MR1**	-0.278917486	0.001462725	-0.23801	0.097427	-0.338701136	0.000505089
**MS4A1**	0.49613521	4.08127E-07	0.588380908	1.54772E-06	0.361314574	0.060322602
**MSR1**	2.090184322	5.50291E-05	2.096634806	0.002799193	2.080756691	0.021319142
**NCF4**	-0.502544188	1.7403E-06	-0.4912643	0.000637919	-0.519030178	0.002249831
**NFATC3**	0.105235214	0.004604782	0.136827971	0.009403209	0.059061	0.496146
**NFIL3**	-0.372637632	0.000643062	-0.35581634	0.026157748	-0.397222603	0.008199687
**NFKB1**	-0.79545124	3.85948E-17	-0.71464798	4.52343E-08	-0.913548312	1.15864E-08
**NFKB2**	-1.035590339	1.0278E-17	-0.93058387	4.52343E-08	-1.189061328	1.18783E-10
**NFKBIA**	-0.929375472	1.81968E-13	-0.8143065	4.30165E-06	-1.097553208	1.15864E-08
**NFKBIZ**	-0.660373378	1.64257E-08	-0.53605548	0.00127915	-0.842068761	3.84915E-06
**NLRP3**	-0.468266914	0.002203625	-0.58871229	0.002656799	-0.292231363	0.285936157
**NOD2**	-0.361212226	0.006838877	-0.34381	0.065951	-0.38664	0.070625
**PAX5**	0.684106563	3.00373E-06	0.810584093	4.23135E-05	0.499254788	0.04754084
**PDCD2**	0.396623252	0.000309816	0.430426776	0.002606424	0.347218	0.098863
**PECAM1**	0.553546251	6.90133E-05	0.471065216	0.009836448	0.674095456	0.004017264
**PLAU**	-1.316292651	1.97984E-08	-1.26929666	0.000140084	-1.384979097	4.02053E-05
**PLAUR**	-0.504355418	0.000365784	-0.57978451	0.001999694	-0.394112905	0.064915461
**POU2F2**	-0.482414564	9.17239E-07	-0.483269	0.000878999	-0.481165777	0.000912509
**PSMB8**	-0.17211	0.030245	-0.04890618	0.722042935	-0.352168114	0.004084143
**PTAFR**	-0.342918864	0.00060921	-0.27654	0.062596	-0.439939362	0.002767448
**PTGER4**	-0.597416412	2.13124E-07	-0.53083817	0.001517981	-0.694723068	4.26947E-05
**PTGS2**	-1.386926469	3.90116E-08	-1.59610243	3.20446E-06	-1.08121	0.015094
**PTPN6**	-0.325569865	0.000282579	-0.25426042	0.043644988	-0.429791368	0.004280161
**PYCARD**	1.130288149	3.07935E-06	1.238654912	0.00127915	0.971905957	0.000825361
**RARRES3**	0.191450911	0.017127959	0.306867286	0.008921978	0.02276544	0.883038304
**RELA**	-0.581464106	9.94774E-09	-0.46431158	0.001127077	-0.752687024	3.84915E-06
**RELB**	-0.432904533	1.93687E-08	-0.3725432	0.000756653	-0.521124937	1.23085E-05
**S1PR1**	0.392213947	7.07808E-07	0.372395484	0.000947654	0.421179392	0.001991995
**sCTLA4**	-0.478750817	0.000730678	-0.44104	0.053153	-0.533867162	0.001850054
**SELL**	0.523442023	1.35938E-10	0.537322904	4.30165E-06	0.503154582	0.000153988
**SELPLG**	0.757833519	8.83505E-10	0.797775589	8.20946E-06	0.699456648	0.000314071
**SERPING1**	0.51739366	0.00164636	0.703850838	0.004768582	0.244879	0.314625

	ALL patients (n=32)		RESPONDERS n=(19)		NON RESPONDERS (n=13)	
**Gene ID**	**logFC**	**adj.P.Val**	**logFC**	**Gene ID**	**logFC**	**adj.P.Val**
**SIGIRR**	0.407921841	1.87018E-08	0.388157515	5.04747E-05	0.436808164	0.001414008
**SLAMF6**	0.416915322	4.31456E-06	0.42569929	0.00214681	0.404077215	0.002437258
**SLAMF7**	-0.854984503	1.03024E-07	-0.59398939	0.00765582	-1.236438906	7.75456E-07
**SMAD3**	-0.457325657	8.20745E-07	-0.40614065	0.002213422	-0.532134521	0.000459663
**SPP1**	-0.794205003	0.006838877	-1.13375815	0.009734778	-0.297935018	0.525169427
**SRC**	-0.670388058	7.48334E-10	-0.63305952	4.9506E-05	-0.724945148	1.44397E-05
**STAT3**	-0.185796453	0.002739462	-0.15686506	0.084910947	-0.22808	0.030486
**STAT4**	-0.187332013	0.009835226	-0.15119	0.181257	-0.240157118	0.033513267
**STAT5A**	-0.511725699	2.11201E-08	-0.3733039	0.004269041	-0.714034475	4.03351E-07
**TBK1**	-0.548140736	3.17416E-09	-0.48200561	0.00013077	-0.644799762	1.23085E-05
**TCF4**	0.240389232	0.000182434	0.253729701	0.005921155	0.220892	0.027591
**TGFBI**	1.257893485	0.000934785	-0.03817041	0.799722111	1.707269505	0.008873181
**TGFBR2**	0.155682235	0.003427715	0.147221	0.045992	0.168048137	0.105730884
**TICAM1**	-0.627297527	9.94774E-09	-0.55943284	0.000251976	-0.726484375	5.38549E-05
**TLR1**	0.301487889	0.003795702	0.336966816	0.012181055	0.249634071	0.194842484
**TLR2**	-0.354026519	0.001753236	-0.38141463	0.010009451	-0.313997736	0.098863245
**TLR7**	1.502003515	4.85702E-07	1.630807897	4.23135E-05	1.313750958	0.008113729
**TLR8**	-0.24383	0.046783	-0.02036	0.923926	-0.570439977	0.000108151
**TMEM173**	0.212835816	0.001818817	0.249259051	0.010675997	0.159602	0.189862
**TNF**	-0.737837176	1.63006E-07	-0.62379687	0.001815452	-0.904511471	5.67222E-05
**TNFAIP3**	-0.743909873	3.73123E-12	-0.67338653	1.74548E-05	-0.846982456	1.5869E-08
**TNFAIP6**	-0.77937602	2.75691E-09	-0.63472702	0.000578627	-0.990786101	4.89881E-07
**TNFRSF10C**	1.662540627	2.75691E-09	1.539185685	5.50216E-05	1.842828618	4.26947E-05
**TNFRSF13C**	0.303073544	0.000626729	0.311564959	0.004009388	0.290663015	0.098863245
**TNFRSF14**	-0.23832668	0.000187472	-0.1966	0.039269	-0.299317481	0.001991995
**TNFRSF1B**	-0.331628502	0.003985162	-0.37665385	0.017275321	-0.26582	0.118605
**TNFRSF8**	-0.537510779	0.000157277	-0.59340591	0.004262181	-0.455817896	0.021041647
**TNFRSF9**	-0.737572652	7.60717E-06	-0.61598	0.012135	-0.915282656	0.00012323
**TNFSF10**	0.489942445	0.001425955	0.627319215	0.006396474	0.289161	0.208458
**TNFSF12**	0.288440067	4.37051E-06	0.244761545	0.009345021	0.352277908	0.000452173
**TNFSF15**	-1.050889668	3.49818E-05	-1.16887786	0.001218513	-0.87844539	0.034916538
**TNFSF8**	-0.564143873	6.42165E-08	-0.57483268	5.4258E-05	-0.548521777	0.002437258
**TRAF1**	-0.861854702	2.3542E-17	-0.81646177	4.52343E-08	-0.928198212	1.15864E-08
**TRAF3**	-0.499738901	6.61848E-10	-0.45991522	8.02238E-05	-0.557942744	3.84915E-06
**XBP1**	-0.566411149	3.14357E-11	-0.50349512	1.72885E-05	-0.65836535	8.47457E-06

*Gene expression data was analyzed in Truculture LPS stimulated samples from 32 patients. Limma analysis was performed to compare gene expression at D0 versus D90 in all 32 patients (column 2 and 3), or selectively in patients classified as Responders (column 4 and 5) or Non-responders, according to ASDAS criteria. Shown are the log fold change and adjusted p-values for the genes that resulted differentially expressed (adjusted p value equal or <0.01) in at least one of the three analyses. The grey shading indicates the comparisons that do not reach statistical significance at the adjusted p-value level of 0.05.

Supplementary Table 7. Gene Module Scoring Table when comparing D0 *vs* D7 for SEB and LPS stimulation

Gene module, SEB stimulation	log fold change	p Value	FDR
NFkB_inhibitors	-1.0811	2.02E-12	9.08E-11
NFkB_transcription_factors	-0.8426	2.51E-11	5.64E-10
NFkB_target_genes	-0.7197	4.46E-11	6.70E-10
TNF	-0.3519	1.23E-08	1.38E-07
NOD	-0.3290	0.0002	0.0009
dectin	-0.3283	4.29E-05	0.0003
IL1	-0.2952	6.18E-05	0.0003
TLR	-0.2914	0.0008	0.0031
NFkB_regulators	-0.2880	1.23E-06	1.11E-05
costimulatory_molecules	-0.2342	3.73E-06	2.8E-05
MAPK	-0.2158	0.0001	0.0007
M1_like_monocytes	-0.1913	0.0359	0.0734
NOTCH	-0.1599	0.0041	0.0142
complement	-0.1530	0.0387	0.0756
type_2_immunity	-0.1468	0.0190	0.0475
adhesion_molecules	-0.1445	0.0118	0.0312
JAK_STAT	-0.1374	0.0299	0.0641
IL6	-0.1241	0.1699	0.2548
autophagy	-0.1139	0.1506	0.2420
innate.immune.response	-0.1131	0.0951	0.1646
allergy	-0.1127	0.3181	0.4469
IFN_targets	-0.1111	0.0813	0.1510
NLR_inflammasomes	-0.1012	0.3928	0.5199
Tregulatory_cells	-0.0950	0.0839	0.1510
cell_cycle	-0.0905	0.1592	0.2470
apoptosis	-0.0806	0.1103	0.1839
phagocytosis	-0.0521	0.4715	0.5894
Tcell_inhibitory_signals	-0.0307	0.6199	0.7153
T_cell_signalling	-0.0052	0.9062	0.9483
type_17_immunity	-0.0037	0.9633	0.9852
TGFB	0.0006	0.9974	0.9974
proinflammatory_molecules	0.0143	0.8390	0.8989
type_1_immunity	0.0235	0.7952	0.8728
Bcells	0.0293	0.6046	0.7153
ubiquitin_proteasome	0.0304	0.5214	0.6341
chemotaxis	0.0324	0.6567	0.7387
lymphopoiesis	0.0458	0.4136	0.5318
MHC_presentation	0.0661	0.3278	0.4469
osteoclast	0.0872	0.3111	0.4469
regulatory_B_cells	0.1668	0.0084	0.0235
NKcells	0.2034	0.0268	0.0604
APC	0.2368	0.0011	0.0041
granulocytes	0.2622	0.0076	0.0229
cytotoxic_molecules	0.2823	0.0268	0.0604
M2_like_monocytes	0.3084	0.0073	0.0229

**Gene module, LPS stimulation**	log fold change	p Value	FDR
NFkB_inhibitors	-1.2400	3.44E-12	3.87E-11
NFkB_transcription_factors	-1.1598	1.78E-15	7.99E-14
NFkB_target_genes	-0.9257	4E-15	8.99E-14
NOD	-0.6677	0.0002	0.0007
IL1	-0.6262	1.47E-08	9.46E-08
TNF	-0.5435	4.41E-13	6.62E-12
M1_like_monocytes	-0.5073	0.0002	0.0006
dectin	-0.4728	0.0003	0.0008
NFkB_regulators	-0.4424	7.69E-10	6.92E-09
costimulatory_molecules	-0.4246	3.5E-08	1.97E-07
MAPK	-0.3875	6.25E-09	4.69E-08
type_1_immunity	-0.3813	0.0374	0.0732
type_2_immunity	-0.3728	0.0006	0.0017
Tregulatory_cells	-0.3393	0.0001	0.0004
type_17_immunity	-0.3152	0.0021	0.0052
TLR	-0.2818	0.0061	0.0144
IL6	-0.2740	0.0002	0.0007
NOTCH	-0.2562	0.0001	0.0004
JAK_STAT	-0.2377	0.0004	0.0011
cell_cycle	-0.2348	0.0001	0.0003
adhesion_molecules	-0.2189	0.0069	0.0156
autophagy	-0.2077	0.0177	0.0379
innate.immune.response	-0.2010	0.0362	0.0732
proinflammatory_molecules	-0.1644	0.1176	0.1864
Tcell_inhibitory_signals	-0.1397	0.0760	0.1368
apoptosis	-0.1290	0.0527	0.0988
complement	-0.1258	0.1201	0.1864
chemotaxis	-0.1028	0.3238	0.4180
ubiquitin_proteasome	-0.0873	0.0977	0.1691
phagocytosis	-0.0541	0.6510	0.7146
IFN_targets	-0.0537	0.5600	0.6300
allergy	-0.0535	0.7086	0.7416
MHC_presentation	-0.0474	0.3977	0.4774
T_cell_signalling	-0.0467	0.1170	0.1864
Bcells	-0.0173	0.7776	0.7952
NLR_inflammasomes	0.0318	0.8381	0.8381
granulocytes	0.0492	0.6799	0.7285
lymphopoiesis	0.0552	0.3251	0.4180
NKcells	0.0682	0.4773	0.5507
osteoclast	0.0878	0.3801	0.4751
M2_like_monocytes	0.1029	0.4031	0.4774
regulatory_B_cells	0.1091	0.1945	0.2917
APC	0.1140	0.2805	0.3944
TGFB	0.1404	0.2162	0.3139
cytotoxic_molecules	0.1906	0.3019	0.4117
